# Linking plant diversity–productivity relationships to plant functional traits of dominant species and changes in soil properties in 15‐year‐old experimental grasslands

**DOI:** 10.1002/ece3.9883

**Published:** 2023-03-08

**Authors:** Peter Dietrich, Nico Eisenhauer, Christiane Roscher

**Affiliations:** ^1^ Department of Physiological Diversity UFZ, Helmholtz Centre for Environmental Research Leipzig Germany; ^2^ German Centre of Integrative Biodiversity Research (iDiv) Halle‐Jena‐Leipzig Leipzig Germany; ^3^ Institute of Biology, Experimental Interaction Ecology Leipzig University Leipzig Germany

**Keywords:** aboveground–belowground, biodiversity effect, biodiversity loss, biodiversity–ecosystem function, functional trait, Jena experiment, plant–soil, soil pH

## Abstract

Positive plant diversity–productivity relationships are known to be driven by complementary resource use via differences in plant functional traits. Moreover, soil properties related to nutrient availability were shown to change with plant diversity over time; however, it is not well‐understood whether and how such plant diversity‐dependent soil changes and associated changes in functional traits contribute to positive diversity–productivity relationships in the long run. To test this, we investigated plant communities of different species richness (1, 2, 6, and 9 species) in a 15‐year‐old grassland biodiversity experiment. We determined community biomass production and biodiversity effects (net biodiversity [NEs], complementarity [CEs], and selection effects [SEs]), as well as community means of plant functional traits and soil properties. First, we tested how these variables changed along the plant diversity gradient and were related to each other. Then, we tested for direct and indirect effects of plant and soil variables influencing community biomass production and biodiversity effects. Community biomass production, NEs, CEs, SEs, plant height, root length density (RLD), and all soil property variables changed with plant diversity and the presence of the dominant grass species *Arrhenatherum elatius* (increase except for soil pH, which decreased). Plant height and RLD for plant functional traits, and soil pH and organic carbon concentration for soil properties, were the variables with the strongest influence on biomass production and biodiversity effects. Our results suggest that plant species richness and the presence of the dominant species, *A. elatius*, cause soil organic carbon to increase and soil pH to decrease over time, which increases nutrient availability favoring species with tall growth and dense root systems, resulting in higher biomass production in species‐rich communities. Here, we present an additional process that contributes to the strengthening positive diversity–productivity relationship, which may play a role alongside the widespread plant functional trait‐based explanation.

## Graphical abstract

We sampled 15‐year‐old plant communities of a biodiversity experiment (Jena Experiment) and tested whether and how changes in plant functional traits and soil properties over time and along the plant species richness gradient contribute to variation in biomass production and biodiversity effects (i.e., net biodiversity, selection, and complementarity effects). We found that changes in soil properties influence plant functional traits, which can explain positive biodiversity effects. Therefore, soil properties and their association with plant functional traits, i.e., plant–soil interactions, should be considered to fully understand the mechanisms driving positive plant diversity–productivity relationships.

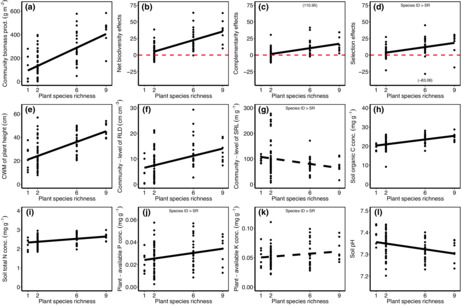

## 1 INTRODUCTION

Positive plant diversity–productivity relationships have been found in many biodiversity experiments and real‐world grasslands (Jochum et al., 2020; Tilman et al., 2001; van der Plas, 2019) highlighting that species‐rich plant communities are more productive than species‐poor communities and that this positive relationship strengthens over time (Cardinale et al., 2007; Guerrero‐Ramirez et al., 2017; Meyer et al., 2016; Reich et al., 2012). Higher productivity of mixtures can be explained by selection and complementarity effects (Cardinale et al., 2007; Fargione et al., 2007; Marquard et al., 2009; Roscher et al., 2007). Positive selection effects emerge from a single or a few highly productive plant species with a disproportionally large effect on community biomass production and the increasing probability for the occurrence of such species in communities with higher species richness (Aarssen, 1997; Huston, 1997). Positive complementarity effects can be induced by niche partitioning or mutualistic interactions, which decrease interspecific competition and thus enhance community biomass production (Loreau & Hector, 2001).

Positive complementarity effects can result from resource partitioning, i.e., species differ in the use of resources, and can exploit resources more completely as a mixture. Furthermore, biotic feedbacks, such as the enhancement of abundance and diversity of mutualistic soil organisms (e.g., arbuscular mycorrhizal fungi [AMF]), can increase complementarity, which decrease interspecific competition and thus enhance community productivity (Barry et al., 2019; Eisenhauer, 2012; Wagg et al., 2011). Next to these two main drivers, there are various other mechanisms, which can enhance complementarity within plant communities, summarized as abiotic facilitation (Barry et al., 2019; Wright et al., 2017); for example, legumes can increase the nutrient availability in soils via symbiotic interactions with rhizobacteria, or plants alter environmental conditions via changes in nutrient availability (Hacker et al., 2015), micro‐climate (Roscher, Kutsch, et al., 2011), or water supply (Guderle et al., 2018).

The number of species per se provides little information about how species interact and function as a community. Therefore, the use of functional traits, which reflect how species acquire resources, has increasingly become an integral part of community ecology (McGill et al., 2006), and trait‐based predictors have been also applied to gain a more mechanistic understanding of the drivers of positive plant diversity–productivity relationships (Hillebrand & Matthiessen, 2009; Roscher et al., 2012). The functional composition of plant communities is determined by the identity of the component species (hereafter: composition effect), the relative abundances of the component species (hereafter: abundance effect), and intraspecific variation in trait expression of individual species (hereafter: adjustment effect; Lepš et al., 2011; Pichon et al., 2022; Roscher, Schumacher, Gubsch, Lipowsky, Weigelt, Buchmann, Schulze, et al., 2018). A classic example of composition effects leading to positive biodiversity effects is when different plant functional groups are present in the community: forbs, legumes, and grasses differ strongly in their leaf and root characteristics, which favors a complementary use of resources, such as light, soil nitrogen, or nutrients, and increase community biomass production (Marquard et al., 2009). Shifts in the abundance of species with particular functional traits may alter plant community biomass production (i.e., abundance effect). For example, it has been shown that plant community biomass production may increase when plant communities become dominated by “fast” species, i.e., species with a high specific leaf area (Pichon et al., 2022). Finally, intraspecific shifts in trait expression in response to growth conditions in plant communities of different diversity may translate into shifts in community trait composition and affect community biomass production (i.e., adjustment effect; Pichon et al., 2022; Roscher, Schumacher, Gubsch, Lipowsky, Weigelt, Buchmann, Schmid, et al., 2018).

In the last few years, particular interest has been given to functional trait‐based approaches. However, there are more potential mechanisms that can additionally explain positive plant diversity–productivity relationships found in long‐term biodiversity experiments. One of the most important influences might be soil properties related to nutrient availability, as it was shown that such abiotic factors modulate the strength of the plant diversity–productivity relationship, demonstrated by a comparison of 26 long‐term biodiversity experiments across Europe and North America (Guerrero‐Ramirez et al., 2017). Moreover, it was found that soil properties change with plant species richness in the long run (Cong et al., 2014; Hacker et al., 2015; Lange et al., 2019; Prommer et al., 2020). For example, soil organic carbon and total nitrogen concentrations increased with plant species richness via increased plant litter input and root exudates (Cong et al., 2014; Prommer et al., 2020), which trigger microbial activity (Lange et al., 2015), affect soil water content (Fischer et al., 2019), and thus the availability of nutrients, such as phosphorus (P) and potassium (K), in soils (Hacker et al., 2015). Moreover, changes in soil nitrogen concentrations can be induced by the presence of particular plant species, nitrogen fixation by legumes, and differences in the use of nitrogen sources (ammonium, nitrate, or organic nitrogen compounds) along the plant species richness gradient (Bessler et al., 2012; Gubsch et al., 2011; Reich et al., 2012). Differences in nitrogen use can also influence soil pH, for example, through the release of inorganic ions by plants when taking up ammonium or nitrate (Hinsinger et al., 2003; Neina, 2019). Other plant‐related processes that can influence soil pH are, inter alia, root exudation, and respiration, or the production of acids by soil microbes through the assimilation of released rhizodeposits (Hinsinger et al., 2003, Neina, 2019). Numerous studies have shown that root exudation and microbial activity (and thus soil organic carbon) increase with higher plant diversity (Eisenhauer et al., 2017; Lange et al., 2015; Mellado‐Vazquez et al., 2016) suggesting that soil pH decreases in species‐rich communities more than in species‐poor communities over time; however, there are no studies that tested this yet. Such a change in soil pH can, in turn, influence numerous processes related to plant growth: one of the most important is the availability and uptake of soil nutrients by plants, as soil pH determines the binding capacity of these nutrients (Devau et al., 2009).

Despite the importance of soil properties, such as nutrient availability and soil pH, for plant productivity, there is a lack of studies that have linked plant diversity‐induced changes in soil properties to community biomass production and biodiversity effects (such as selection and complementarity) in long‐term biodiversity experiments (Guerrero‐Ramirez et al., 2017). Moreover, it is not known whether changes in soil properties may influence community biomass production and biodiversity effects directly, or indirectly via soil‐induced changes in plant functional traits. To fill these knowledge gaps, we investigated 15‐year‐old plant communities of a grassland biodiversity experiment, which consisted of nine potentially dominant species (Dominance Experiment, a sub‐experiment of the Jena Experiment established in 2002; Roscher et al. (2004)). The experiment included five grass species, two forb species, and two legume species. For our study, we used plant communities with 1, 2, 6, and 9 plant species. We measured plant biomass production and used it to calculate biodiversity effects according to the additive partitioning method by Loreau and Hector (2001). Moreover, we determined plant functional traits, which are known to be related to resource acquisition and use (plant height, SLA, leaf N, P, K; Roscher et al. (2012)), and AMF colonization rates, as a proxy for biotic feedbacks between plants and soil mutualists, to test how the community means of these traits influence biomass production and biodiversity effects. We applied the variance partitioning method following Lepš et al. (2011) to test whether changes in the community‐weighted means (CWM) of these traits along the plant species richness gradient were caused by different species compositions (composition and abundance effects) and/or intraspecific trait variation (adjustment effects). Finally, to test whether and how soil properties related to nutrient availability influence community biomass production and biodiversity effects in the 15‐year‐old plant communities, we determined soil pH, organic carbon (C), total nitrogen (N), plant‐available phosphorus (P), and potassium (K) concentrations. We hypothesized that

1. plant species richness and the presence of particular dominant plant species increase community biomass production and biodiversity effects.
2. CWM of plant traits and AMF colonization rates change with plant species richness and species identity, whereby composition and abundance effects, as well as intraspecific shifts in trait expression (adjustment effects), play a role for this change.
3. soil organic C and total N increase with plant diversity and the presence of dominant species (due to enhanced root exudation and microbial activity). This in turn causes a stronger reduction in soil pH in species‐rich than in species‐poor communities over time lowering the binding capacity of nutrients. Consequently, the availability of P and K increases with plant diversity.
4. that soil property changes along the plant species richness gradient affect biomass production and biodiversity effects directly, as well as indirectly via soil‐induced changes in plant functional traits.

## 2 MATERIALS AND METHODS

### 2.1 Study design

This study was carried out in the Jena Experiment, which is a long‐term grassland biodiversity experiment (Roscher et al., 2004). The study site is located in the floodplain of the Saale river near the city of Jena (Thuringia, Germany, 50°55′ N, 11°35′ E, 130 m.a.s.l.) and had been used as a high‐fertilized arable field for growing wheat and vegetables until the biodiversity experiment was established in 2002. The soil is a Eutric Fluvisol, while soil texture changes from sandy loam to silty clay with increasing distance from the river on the experimental site. The study site was divided into four blocks to account for differences in soil texture, while blocks were arranged parallel to the riverside (Roscher et al., 2004). The mean annual air temperature was 9.7°C and the mean annual precipitation was 574 mm from 2003 to 2016, which was recorded with a meteorological station at the study site (Weather Station Jena‐Saaleaue, Max Planck Institute for Biogeochemistry Jena, https://www.bgc‐jena.mpg.de/wetter/).

For the present study, the Dominance Experiment was used, which was a sub‐experiment of the Jena Experiment (Roscher et al., 2004). The species pool of this experiment consisted of nine plant species, which often reach dominance in Central European mesophilic grasslands of the Arrhenatherion type (Ellenberg, 1988): five grass species (*Alopecurus pratensis* L., *Arrhenatherum elatius* (L.) P. Beauv. ex J. Presl et C. Presl, *Dactylis glomerata* L., *Phleum pratense* L., *Poa trivialis* L.), two legume species (*Trifolium pratense* L., *T. repens* L.), and two forb species (*Anthriscus sylvestris* (L.) Hoffm., *Geranium pratense* L.). Species richness levels ranged from one to nine species (1, 2, 3, 4, 6, and 9 plant species plots), while each species and every species pair occurred the same number of times at each species richness level. All species compositions were replicated twice (i.e., same mixture identity), with the exception of the nine‐species mixture, which was replicated eight times. There was the same number of plots per species richness level in each block, ensuring that replicates with identical species composition were distributed in different blocks. From the year of establishment (2002) until 2009, plants were grown in plots of 3.5 × 3.5 m, while plot size was reduced to 1 × 1 m in 2010. Seeds for the establishment of the experiment were purchased from a commercial supplier (Rieger‐Hoffman GmbH) and were sown in May 2002 with a density of 1000 viable seeds per m^2^. One species, *A. sylvestris*, which failed to establish in the first growing season, was re‐sown with half density in late autumn 2002 (Roscher et al., 2004), while no further re‐sowing was done later. All plots were mown every year in June and September (mown plant material was removed), were regularly weeded to maintain the sown species compositions, and have never received any fertilizer.

To keep the number of samples and measurements manageable, we used the 1‐, 2‐, 6‐, and 9‐species plots of the Dominance Experiment (85 plots out of 206 plots). Due to very low amounts of standing biomass in some monocultures, we decided to carry out destructive measurements in only one of the two monocultures per species (with the exception of aboveground biomass, which was measured in both monocultures), so that the other can still be sampled in the future. Furthermore, the monocultures of the grass species *P. pratense* and the forb species *A. sylvestris* showed no biomass production in 2016/2017, and both species were extinct or had a very low biomass in all other plots, so that we did not sample these monoculture plots and did not measure any functional traits of these two species in mixtures (i.e., these two species were excluded from the analyses). For all remaining plant species, we conducted measurements in each of the seven monoculture plots, a subset of the two‐species mixtures (= 46 plots), and all 6‐ and 9‐species plots (= 24 and 8 plots, respectively; Table 1). In case of the two‐species mixtures, we used all existing two‐species combinations of the seven species (both replicates), and one replicate with *A. sylvestris* and *P. pratense*, respectively, although there are some exceptions due to local extinctions (Table S1). Overall, each of the seven species was present nine to 12 times in the two‐species plots (for detailed information see Table S1), 16 times in the six‐species plots, and eight times in the nine‐species plots. Because of the extinction of several plant species, we counted how many of the originally sown plant species were actually growing in the plots in May 2017 and used this “realized plant species richness” as another explanatory variable, in addition to sown plant species richness.

**TABLE 1:** Summary table of the seven plant species used in the experiment and their characteristics: biomass production, proportion of biomass to total community biomass, plant functional traits, and AMF colonization rates.

	Biomass production		Plant functional traits					
	Biomass production (g m^−2^)	Proportion (%)	Plant height (cm)	Specific leaf area (mm^2^ mg^−1^)	Leaf N concen. (mg g^−1^)	Leaf P concen. (mg g^−1^)	Leaf K concen. (mg g^−1^)	AMF colonization rates (%)
Grass species								
*Arrhenatherum elatius*	276.4 ± 132.6	78.7 ± 19.8	45.6 ± 6.1	24.4 ± 2.5	23.7 ± 3.4	3.6 ± 0.6	25.5 ± 3.0	40.6 ± 13.8
*Alopecurus pratensis*	66.2 ± 62.8	30.4 ± 38.1	29.0 ± 7.7	17.1 ± 3.1	17.7 ± 2.9	2.8 ± 0.4	23.8 ± 2.4	30.7 ± 12.7
*Dactylis glomerata*	40.8 ± 43.3	34.1 ± 39.6	23.7 ± 6.6	23.3 ± 2.8	22.0 ± 4.0	3.3 ± 0.5	26.3 ± 3.8	33.5 ± 17.0
*Poa trivialis*	20.2 ± 23.1	21.2 ± 33.4	12.5 ± 4.2	29.7 ± 6.2	16.1 ± 3.6	2.6 ± 0.7	27.8 ± 4.8	11.0 ± 6.1
Forb species								
*Geranium pratense*	73.5 ± 52.0	33.6 ± 32.1	27.9 ± 7.6	18.2 ± 1.8	26.3 ± 3.4	4.2 ± 0.6	14.2 ± 3.3	65.5 ± 14.9
Legume species								
*Trifolium pratense*	46.6 ± 67.7	22.7 ± 37.5	15.2 ± 5.2	25.5 ± 4.2	40.7 ± 5.0	2.5 ± 0.3	10.2 ± 3.3	77.6 ± 9.7
*Trifolium repens*	2.1 ± 1.4	5.9 ± 23.5	11.9 ± 3.0	19.9 ± 1.0	41.3 ± 5.7	2.6 ± 0.7	8.7 ± 2.7	86.5 ± 10.6

*Note*: Shown are means ± standard deviation across all plots, where the respective species were sown.

### 2.2 Plant‐related measurements

In May 2017, three plants per species and plot (if possible) were selected and plant height (cm) was measured as the stretched length of three vegetative shoots per individual. First, the heights of the three shoots per individual were averaged and then the mean values of the three individuals per plot. After that, bulk samples of 10–15 fully developed leaves were collected from the same individuals and shoots (one to three leaves per shoot). Leaves were stored in sealed plastic bags in a cooling box for transport to the laboratory, where leaf area (mm^2^_leaf_) was measured with a leaf area meter (LI‐3000C Area Meter equipped with LI3050C transparent belt conveyor accessory; LI‐COR). Then, leaf samples were dried for 48 h at 70°C, weighed, and specific leaf area (SLA; mm^2^_leaf_ mg^−1^_dw_) was calculated as the ratio between total leaf area and total leaf mass per plot and species. Dry leaf samples were ground to a fine powder with a mixer mill (MM2000, Retsch). Approximately 10 mg of the milled material was then used to determine leaf nitrogen concentration (mg N g^−1^_leaf_) with an elemental analyzer (Vario EL cube, Elementar Analysensysteme GmbH). Leaf phosphorus (mg P g^−1^_leaf_) and leaf potassium concentration (mg K g^−1^_leaf_) were measured using an inductively‐coupled plasma optical emission spectrometer (Thermo Scientific™ iCAP™ 7400 ICP‐OES Duo). Therefore, milled leaf samples (250 mg) were first treated in a Mars 6 microwave closed system (CEM GmbH) for acid digestion (with 5 mL of HNO_3_ and 0.5 mL of H_2_O_2_) and then the diluted acid extracts were analyzed with the ICP‐OES to measure P and K.

For the determination of root colonization by arbuscular mycorrhizal fungi (AMF), we collected roots of the selected plant individuals by taking soil cores (10 cm depth, 5 cm diameter), which contained the root crown and attached roots of the plants. Soil was roughly removed and roots per plot and species were stored in plastic bags. In the laboratory, roots were cleaned by rinsing off the remaining soil with tap water, and then the material was stored in 70% ethanol until further processing. For the determination of AMF colonization, roots were first rinsed with tap water to remove ethanol, and then, a subsample of ~20 g of finer roots was purified by heating in 10% potassium hydroxide solution at 80°C for 30–90 min (heating times varied depending on plant species). After this, roots were heated for 5 min at 80°C in an ink–vinegar solution (5% black ink: Parker S0037460 Quink Black; 95% vinegar: white household vinegar, 5% acetic acid) to stain AMF following Vierheilig et al. (1998). After staining, roots were rinsed several times with and stored in a water‐vinegar mixture to remove excess stain. Finally, AMF colonization was scored under the microscope (200x magnification) using the line‐intersect method for 100 intersects (McGonigle et al., 1990).

For the determination of community‐level root traits, we took two soil cores (10 cm depth, 5 cm diameter) per plot in June 2017 in the inner center, i.e., with a distance of at least 30 cm from the plot edge. Soil cores were pooled per plot and stored in a freezer until further analysis (−20°C). Later, soil cores were defrosted, and roots were cleaned with tap water. Then, root samples per plot were scanned with a flatbed scanner at 800 dpi (Epson Expression 10000 XL scanner, Regent Instruments), and root length was measured with an image analysis software (WinRHIZO; Regent Instruments), followed by drying (at 70°C for 48 h) and weighing. Specific root length (SRL) was calculated as the ratio between root length and root dry mass (m_root_ g_root_^−1^), and root length density (RLD) as the ratio of root length to volume of the soil cores (cm_root_ cm_soil_^−3^).

Aboveground biomass was harvested block‐wise on each plot from 29 May to 5 June 2017. A sample area of 0.2 × 0.5 m was chosen in the inner center of the plots, and plants were cut 3 cm above ground. Biomass samples were sorted to sown plant species, weeds, and dead plant material, dried at 70°C for 48 h, and weighed. Total aboveground biomass of the sown plant species per plot was extrapolated to one square meter (g m^−2^) as a measure of community biomass production.

### 2.3 Soil‐related measurements

For the determination of soil properties, we took three soil samples (10 cm depth, 2.5 cm diameter) in May 2017 in the inner center of the plots. Soil samples were pooled per plot, sieved to 2 mm, and then air‐dried. In a subsample, plant residues (root fragments, etc.) were first removed with tweezers, then this sample was ground to a fine powder with a mixer mill (MM2000, Retsch), dried for 5 h at 40°C, and soil total nitrogen (N) and total carbon (C) concentrations were analyzed with an elemental analyzer (Vario EL cube, Elementar Analysensysteme GmbH). For the determination of soil organic carbon concentration, soil carbonate was measured volumetrically with a calcimeter according to Scheibler (Schlichting & Blume, 1966) and subtracted from total carbon concentrations. The other part of the soil sample was used for the determination of plant‐available phosphorus (P) and potassium (K) concentrations, as well as soil pH. For the determination of phosphorus concentration after the Olsen P method (Olsen, 1954), soil was extracted with 0.5 M sodium hydrogen carbonate solution (pH 8.5) using the phosphomolybdate blue method (Murphy & Riley, 1962). Plant‐available P was measured in the solution with a plate reader (Varioskan LUX, Thermo Electron LED GmbH, Osterode am Harz, Germany). To determine potassium concentration, soil was extracted with 1 M calcium‐acetate‐lactate and plant‐available K was measured with the ICP‐OES (Thermo Scientific™ iCAP™ 7400 ICP‐OES Duo). Finally, soil pH was determined in a 0.01 M calcium chloride suspension with a pH Meter (pH Meter 766, Knick, Berlin, Germany).

### 2.4 Data analyses

To test for overyielding, i.e., whether plant mixtures produce more biomass relative to the biomass production of the same species in monoculture, and whether this overyielding is caused by the presence of a high‐productive plant species (selection effects [SEs]) or by niche differentiation and facilitative interactions among species (complementarity effects [CEs]; Loreau, 1998), we used the additive partitioning method by Loreau and Hector (2001):

$${\mathrm{SE}}_{i}=\left(∆{\mathrm{RY}}_{i}-∆\bar{\mathrm{RY}}\right)\times \left(M_{i}-\bar{M}\right)$$

$${\mathrm{CE}}_{i}=M_{i}\times ∆{\mathrm{RY}}_{i}-\left(∆{\mathrm{RY}}_{i}-∆\bar{\mathrm{RY}}\right)\times \left(M_{i}-\bar{M}\right)$$

where *ΔRY*_*i*_ is the deviation from the expected relative yield of species *i* in the mixture (RY_observed_‐RY_expected_), and *M*_*i*_ is the yield of species *i* in monoculture. For the calculation of CEs, SEs, and net biodiversity effects (NEs), with the latter being the sum of SEs and CEs and representing overyielding, we used for each species the averaged biomass of the sampled monoculture and its identical replicate, to account for the location of the plots in different blocks. To test whether biodiversity effects were larger than zero (which would indicate the overyielding of plant mixtures compared with monocultures), we used analyses of variance (ANOVA) with block, sown plant species richness, and mixture identity, in order to test grand means against hypothetical values (i.e., their deviation from zero, respectively).

For plant traits, which were measured at the species level (plant height, SLA, leaf nutrient concentrations, AMF colonization rates), we calculated community‐weighted means (CWM) per plot. CWM is the mean trait value weighted by species' relative abundances according to the equation:

$$\mathrm{CWM}=\sum_{i=1}^{n}p_{i}t_{i}$$

where *n* is the number of species in the community, *p*_*i*_ is the species biomass proportions, and *t*_*i*_ is species‐specific trait values in the respective plot. Furthermore, we tested whether changes in CWM along the species richness gradient were caused by different species compositions and abundances (= composition and abundance effects), or intraspecific trait variation as a result of an adjustment to the changing environment (= adjustment effects). To disentangle composition/abundance effects from adjustment effects, we followed the variance partitioning method proposed by Lepš et al. (2011). For the calculation of composition/abundance effects, we used the same equation as for the calculation of CWM, but instead of entering species‐specific trait values per plot for *t*_*i*_, we used species trait values averaged across all plots. Adjustment effects were then calculated by subtracting composition/abundance effects from CWM per plot (adjustment effect = CWM – composition/abundance effects). Finally, we used decomposition of the total sum of squares from ANOVAs with the sequence block, plant species richness, and mixture identity as explanatory variables; and CWM, composition/abundance effect, and adjustment effect values, respectively, as response variables to determine the proportion of variance explained by plant species richness.

To test hypotheses H1, H2, and H3 whether plant species richness and plant species identity influence community biomass production, biodiversity effects (NEs, SEs, CEs), plant traits (community‐level RLD and SRL, and CWM of the other plant traits), and soil properties, we used linear mixed‐effects models. We started with a null model with the random effects block and mixture identity only, and then extended the model stepwise by adding sown plant species richness (or realized plant species richness) and presence/absence of particular plant species (= plant species identity; for each plant species separately) as fixed effects (= “model 1”).

Model1:y∼plant species richness+plant specis identity+(1|block)+(1|mixture identity)

where *y* is either community biomass production, NE, SE, CE, CWM for the different measured plant traits, or soil properties, respectively. Because plant species richness and plant species identity were not completely independent, we also tested the reversed sequence, i.e., we added first plant species identity and then sown plant species richness (=“model 2”).

Model2:y∼plant specis identity+plant species richness+(1|block)+(1|mixture identity)

We only considered plant species identity to be significant if it was significant in both types of models. Furthermore, if plant species richness had a significant effect in model 1 but not in model 2 (when fitted after species identity), this indicates that the presence of the respective plant species was responsible for the species richness effect (in these cases, plant identity had in both models a significant influence). Mixed‐effects models were fitted with maximum likelihood (ML), and likelihood ratio tests were used to compare models and assess the significance of the fixed effects.

Moreover, we used a correlation matrix and standardized principal component analysis (PCA; first principal component [PC1] and second principal component [PC2]) to test for relationships between plant and soil variables, among and with each other. We calculated a PCA with plant functional traits only (“plant PCA”), a PCA with soil properties only (“soil PCA”), and a PCA with both types of variables (“plant+soil PCA”). Finally, PCA was used to check how plots with different levels of plant species richness (1, 2, 6, 9) and with different proportions of sown grass species (0%, 33%–50%, 67%–100%; for plant + soil PCA only) are distributed within the multivariate space.

To test hypothesis 4 whether plant and/or soil variables can predict community biomass production and biodiversity effects, we used the same mixed‐effects model approach as described above, with productivity‐related variables as response, block and mixture identity as random effects, and plant and soil variables as fixed effects, in separate models.

$$\mathrm{Model}\;3:y_{\mathrm{biomass}}∼\mathrm{one of the measured plant or soil variables}\;/\;\mathrm{PC}1\;scores\;\mathrm{or}\;\mathrm{PC}2\;\mathrm{scores}+\left(1|\mathrm{block}\right)+\left(1|\mathrm{mixture identity}\right)$$

where *y*_biomass_ is community biomass production or one of the biodiversity effects (NEs, SEs, CEs), respectively. To check for direct and indirect effects of plant and soil variables on biomass production and biodiversity effects, we applied piecewise structural equation modeling (SEM). We started with an initial model for biomass production and biodiversity effects, respectively, containing plant species richness, the presence of *A. elatius* (as the species with the strongest effect on almost all variables), plant PC1 and PC2 scores, as well as soil PC1 and PC2 scores derived from the plant and soil PCAs (the initial model can be found in the Figure S1). We decided to use PC scores and only *A. elatius* in order to avoid that the complexity of the SEMs becomes too large. Furthermore, by taking the PC scores, we eliminated the collinearity of many of the measured plant and soil variables. Piecewise SEMs were based on mixed‐effects models accounting for block and mixture identity as random effects, as it was done in all previous mixed‐effects models. Model fit was assessed using Fisher's C statistic, where *p* > .05 indicates that the data are well represented by the model. Finally, we used variance partitioning to test how much variance in community biomass production and biodiversity effects is explained by plant traits, soil properties, and plant species richness, individually and combined. Therefore, we constructed a model for each productivity‐related variable containing three groups of predictors: plant traits, i.e., plant PC1 and PC2, soil properties, i.e., soil PC1 and PC2, and species richness (log‐transformed, as for LMM analyses).

Prior to all these analyses, variables were transformed to meet the assumptions of normality and variance homogeneity: community biomass production, RLD, and SRL were square‐root‐transformed, and NEs, SEs, and CEs were square‐root‐transformed with sign reconstruction (sign(y) = |y|) (Loreau & Hector, 2001). Moreover, we removed one plot (two‐species plot with *D. glomerata* and *A. sylvestris*) from some analyses, because of missing values of leaf P and K. All analyses were performed with the statistical software R (version 3.6.1, R Development Core Team, http://www.R‐project.org). For linear mixed‐effects models, we used the *lmer* function in the R package *lme4* (Bates et al., 2015), for PCA the *rda* function and for variance partitioning the *varpart* function of the R package *vegan* (Oksanen et al., 2007), and for SEMs the function *psem* of the R package *piecewiseSEM* (Lefcheck, 2016).

## 3 RESULTS

### 3.1 Plant species richness and species identity influence plant community biomass production and biodiversity effects (H1)

**FIGURE 1:**
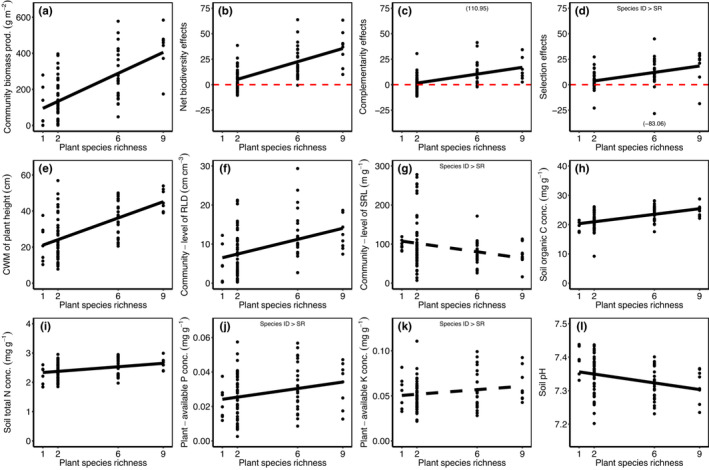
Relationships between plant species richness and community biomass production (a), net biodiversity effects (b), complementarity effects (c), selection effects (d), CWM of plant height (e), community‐level root length density (f), community‐level specific root length (g), soil organic carbon concentrations (h), soil total nitrogen concentrations (i), plant‐available phosphorous concentrations (j), plant‐available potassium concentrations (k), and soil pH (l). Each dot represents a plant community. Solid black lines indicate significant relationships between plant species richness and variables, and dashed black lines indicate marginal significant relationships. The phrase “Species ID > SR” indicates that the (marginal) significant relationship found in the mixed‐effect model, where species richness was fitted first (“model 1”), was neutralized by the species ID effect in the model, where species ID was fitted before species richness (“model 2”; see Table 2). Red dashed lines in panels (b), (c), and (d) show the borderline to positive biodiversity effects (NEs/SEs/CEs > 1). Note that two data points were excluded from (c) and (d) because they have either very high positive or negative values. These data points are indicated as text in brackets.

Community biomass production and biodiversity effects (NEs, CEs, SEs) increased with plant species richness (Table 2; Figure 1a–d). Additionally, community biomass production and NEs were greater in communities with *A. elatius*, and CEs were positively affected by the presence of *P. trivialis* (Table 2; Table S2). In case of SEs, sown plant species richness was not significant anymore, when we fitted the presence of *A. elatius* before species richness (Table 2; Table S2). Biodiversity effects were significantly higher than zero (NEs: *F*_1,42_ = 100.09, *p* < .001; CEs: *F*_1,42_ = 23.01, *p* < .001; SEs: *F*_1.42_ = 15.26, *p* < .001) across all species richness levels. Overall, using sown plant species richness or realized plant species richness revealed similar results indicating no bias due to species extinction (see Tables S2–S5; Figure S2).

**TABLE 2:** Summary of mixed‐effect model analyses testing the effects of sown plant species richness and plant species identity on community biomass production, biodiversity effects, community means of plant traits, and soil properties.

	Plant species richness (SR)			*Arrhenatherum elatius*	*Alopecurus pratensis*	*Dactylis glomerata*	*Poa trivialis*	*Geranium pratense*	*Trifolium pratense*	Neutralization of SR effect?
	*χ* ^2^	*p*								
Production variables										
Community biomass	24.54	**<.001**	+	+						No
Net biodiversity effects	25.23	**<.001**	+	+						No
Selection effects	5.11	**.024**	+	+						Yes
Complementarity eff.	21.16	**<.001**	+				+			No
Plant traits										
Plant height	13.07	**<.001**	+	+						No
Specific leaf area (SLA)	1.14	.286		+			+			
Leaf N concentration	0.33	.564							+	
Leaf P concentration	0.58	.446		+			−			
Leaf K concentration	0.82	.364		+					−	
Root length density (RLD)	10.41	**.001**	+	+	+					No
Specific root length (SRL)	3.75	*.053*	−					−		Yes
AMF colonization rate	0.26	.610				−	−			
Soil properties										
Soil organic carbon con.	19.25	**<.001**	+	+						No
Soil N concentration	11.61	**<.001**	+	+						No
Soil P concentration	4.45	**.035**	+	+		+				Yes
Soil K concentration	3.52	*.060*	+	+						Yes
Soil pH	14.52	**<.001**	−	−						No

*Note*: Columns 2–4 (“Plant species richness (SR)”) indicate the results of mixed‐effects model analysis with plant species richness as the first fixed effect (i.e., model 1). Shown are degrees of freedom (df), Chi^2^, and *p*‐values (*p*). Significant effects (*p* < .05) are given in bold and marginally significant effects (*p* < .10) in italics. The plus‐icon behind *p*‐values indicates a significant increase, while a minus‐icon indicates a decrease in the variable with species richness. The plus‐ and minus‐icons in the remaining columns imply, whether the presence of a specific plant species positively or negatively influenced the response variables (requirement: the species ID effect was significant in both models: for model 1 when species ID was fitted after species richness, and for model 2, when species ID was fitted before species richness; the full results can be found in Tables S2–S5). The last column provides information on whether the (marginal) significant species richness effect found in model 1 (see columns 2–4) was neutralized by the species ID effect in model 2 (when species ID was fitted before species richness). Note that we removed the legume *T. repens* from the list, as it had no effect at all and that degrees of freedom (DF) was one for all variables fitted in the models.

### 3.2 Plant species richness and species identity influence community‐weighted means (CWM) of plant traits and AMF colonization rates (H2)

Two plant trait variables increased along the plant species richness gradient: CWM of plant height and community‐level root length density, while the CWM of other plant traits did not change with plant species richness (Table 2, Figure 1e,f; Tables S3 and S4). The presence of particular plant species increased several trait means: CWM of plant height was increased by the presence of *A. elatius* (next to plant species richness), CWM of SLA by the presence of *A. elatius* or *P. trivialis*, CWM of leaf N by the presence of *T. pratense*, and community‐level RLD by the presence of *A. pratensis* or *A. elatius* (next to plant species richness). On the contrary, CWM of AMF colonization rates was significantly decreased by the presence of *D. glomerata* or *P. trivialis* (Table 2; Tables S3 and S4). In case of leaf P and K, the presence of *A. elatius* increased their CWM, while the presence of *P. trivialis* decreased CWM of leaf P, and *T. pratense* decreased CWM of leaf K (Table 2; Table S3). The marginally significant influence of plant species richness on community‐level specific root length (negative relationship; Figure 1g) disappeared when the presence of *G. pratense* was fitted first in the model (Table 2; Table S4).

**FIGURE 2:**
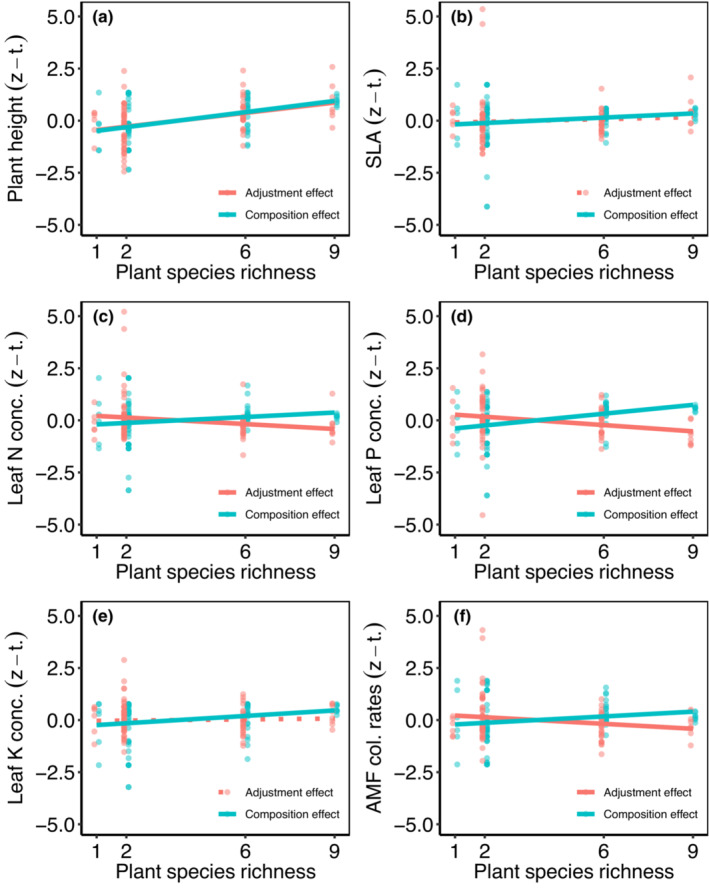
Relationships between plant species richness and composition/adjustment effects of plant height (a), specific leaf area (SLA; b), leaf nitrogen (N) concentration (c), leaf phosphorous (P) concentration (d), leaf potassium (K) concentration (e), and AMF colonization rates (f). Each circle represents the value for one plant community (red = adjustment effect, turquoise = abundance/composition effect), red lines indicate relationships between species richness and adjustment effects and turquoise lines relationships between species richness and abundance/composition effects. Solid lines indicate significant relationships, and dotted lines indicate nonsignificant relationships. Adjustment and abundance/composition effects (different scales) were z‐transformed to compare them.

Variance partitioning of plant species richness effects on CWM revealed that species richness explained 39% of the variance in plant height, while for the other traits, species richness only explained between 1% and 10% (Tables S6 and S7). For plant height, variation explained by plant species richness was mainly caused by composition/abundance effects (19%) and the interaction of composition/abundance and adjustment effects (17%), while adjustment effects alone explained only 4% (Table S7). In line with this, we found that composition and adjustment effects significantly increased with plant species richness (Figure 2a; Table S6). In case of SLA and leaf K, we found that composition/abundance effects explained 5% of the variance, respectively, adjustment effects explained 1% and 0%, and interaction of both effects 4% and 1%, resulting in a total proportion of variance of 10% and 6% explained by plant species richness (Table S7). We found an increase in composition/abundance effects with plant species richness but no significant change in adjustment effects along the plant species richness gradient for SLA and leaf K (Figure 2b,e; Table S6). For leaf N, leaf P, and AMF colonization rates, composition/abundance effects explained 2%, 16%, and 2% of the variance, adjustment effects explained 4%, 7%, and 5%, respectively; however, the interactive impacts on both effects were negative (−6%, −21%, and −6%, respectively; Table S7). Negative interaction effects were caused due to the fact that composition/abundance effects significantly increased with plant species richness, while adjustment effects significantly decreased (Figure 2c,d,f; Table S6), explaining the overall low proportion of variance explained by plant species richness for leaf N, leaf P, and AMF colonization rates.

### 3.3 Plant species richness and species identity influence soil properties (H3)

Soil organic carbon, total nitrogen, and plant‐available P and K significantly increased, and soil pH decreased with plant species richness (Table 2, Figure 1h–l; Table S5). In addition to the influence of plant species richness, the presence of *A. elatius* increased soil organic carbon and total nitrogen and decreased soil pH (Table 2; Table S5). In case of plant‐available P and K, the positive effect of plant species richness disappeared, when we fitted *A. elatius* or *D. glomerata* as the first fixed effect in the models for P (in separate models), and *A. elatius* in the models for K (Table 2; Table S5).

### 3.4 Relationships between plant traits and soil properties (PCA results)

**FIGURE 3:**
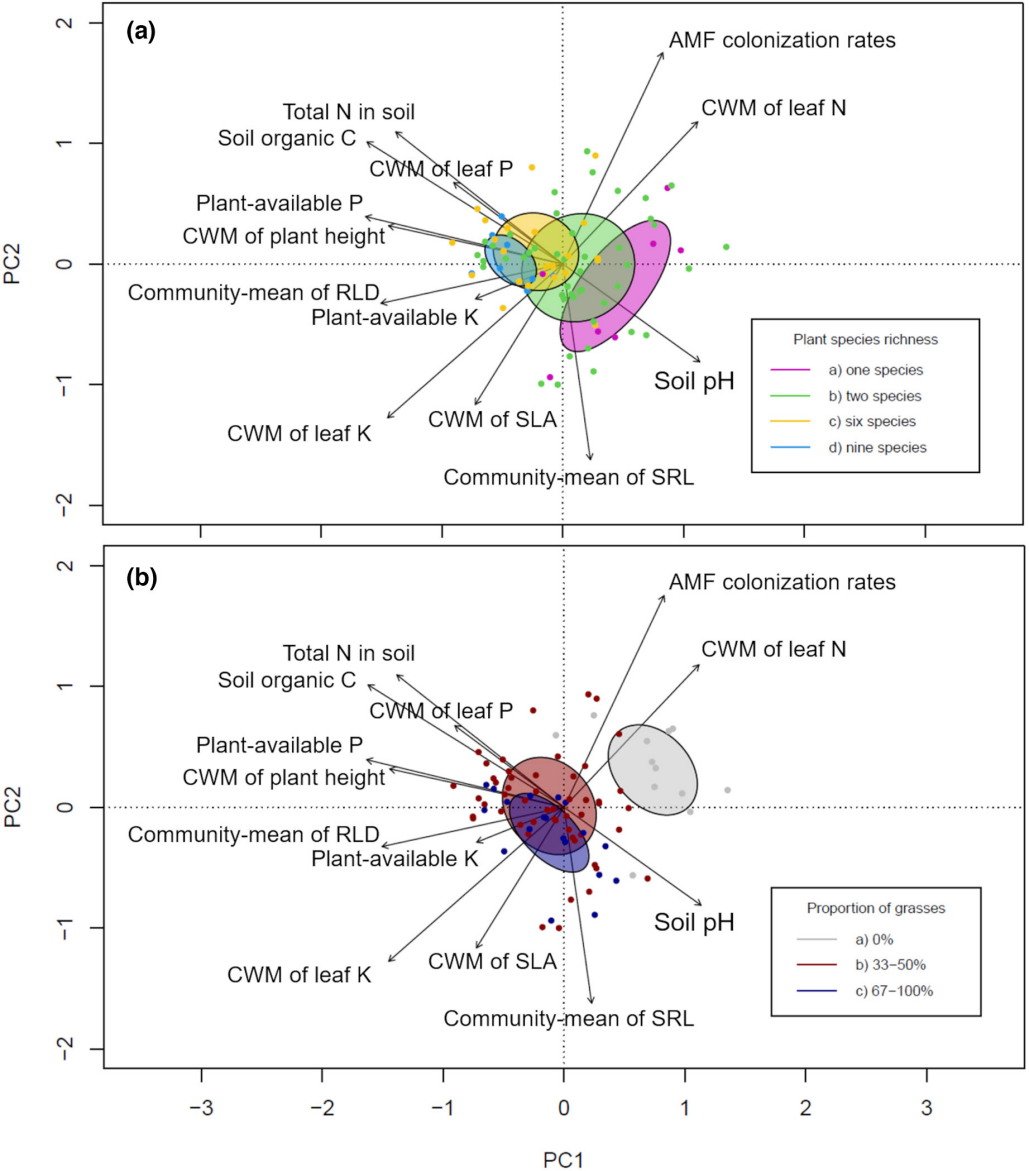
Standardized principal components analysis (PCA; first vs. second axes) of 84 plant communities characterized by eight plant variables (community‐level root length density [RLD] and specific root length [SRL], and CWM of plant height, specific leaf area [SLA], leaf nitrogen [N], leaf phosphorus [P] and leaf potassium [K] concentrations, and AMF colonization rates) and five soil variables (concentrations of organic carbon [C], total nitrogen [N], plant‐available phosphorus [P] and plant‐available potassium [K], soil pH). Shown are sown plant species richness groups (a) and community groups differing in proportion of grass species (b) as ellipses indicating the standard deviation of point scores for each group (a: 1, 2, 6, and 9 plant species; b: communities with 0%, 33%–50%, and 67%–100% grass species). Each dot represents a plant community, different colors indicate the affiliation to the groups.

The two leading axes of the PCA including plant traits and soil properties (plant+soil PCA, Figure 3) explained about 50.5% of the total variation. The first principal component (PC1) accounted for 32.7% of the variance, and the second principal component (PC2) for 17.8%. Plant species richness levels were separated along a sequence extending from bottom right to top left (Figure 3a). Species‐poor communities were characterized by high SRL and soil pH while species‐rich communities by high CWM of plant height, soil organic C, total N, and plant‐available P (Figure 3a). Moreover, another sequence extending from top right to bottom left separated the communities with and without grass species (Figure 3b). Communities without grass species were characterized by high CWM of AMF colonization and leaf N, while communities with grass species showed high CWM of SLA and leaf K (Figure 3b). In case of the PCA including only plant traits (plant PCA), the first PC explained 37.3% and the second PC 21.2% (in total 58.5%) of variation (Figure S3). Plant PC1 had high negative loadings for CWM of leaf K, RLD, and plant height, and high positive loadings for CWM of leaf N and AMF colonization rates. Plant PC2 had high negative loadings for SRL and high positive loadings for plant height and leaf P. The first two axes of the soil PCA (PCA including only soil properties) explained 77.3% of variation, while soil PC1 explained 53.5% and soil PC2 23.8% (Figure S4). Soil PC1 had high negative loadings for soil organic carbon, soil N, and soil P concentrations, and a high positive loading for soil pH. Soil PC2 had high negative loadings for soil K and soil pH, while there were no variables causing high positive loadings. Correlation matrix results can be found in the Appendix S1 (Tables S8 and S9; Appendix Section S1).

### 3.5 Direct and indirect effects of plant and soil variables on community biomass production and biodiversity effects (H4)

Mixed‐effects model analyses showed several significant effects of plant traits, soil properties, and PCs on productivity‐related variables, which can be found in Table 3.

**TABLE 3:** Summary of mixed‐effect model analyses testing the effects of plant and soil variables (as single variables and condensed as scores [PC1 and PC2] derived from principal component analysis) on community biomass production and biodiversity effects.

	Community biomass			Net biodiversity effects			Selection effects			Complementarity effects		
	*χ* ^2^	*p*		*χ* ^2^	*p*		*χ* ^2^	*p*		*χ* ^2^	*p*	
Plant traits												
PC1 (leaf K → leaf N)	10.93	**<.001**	−	0.77	.381		5.93	**.015**	−	<0.01	.980	
PC2 (SRL → height)	14.46	**<.001**	+	1.12	.290		2.28	.131		0.09	.769	
Plant height	85.93	**<.001**	+	15.47	**<.001**	+	13.44	**<.001**	+	5.14	**.023**	+
Specific leaf area (SLA)	0.99	.320		3.03	*.082*	+	1.21	.271		5.60	**.018**	+
Leaf N concentration	0.31	.576		0.09	.770		1.58	.209		2.52	.113	
Leaf P concentration	0.93	.334		0.28	.597		3.12	*.077*	+	2.20	.138	
Leaf K concentration	2.06	.151		1.00	.317		5.84	**.016**	+	4.86	**.027**	−
Root length density (RLD)	27.55	**<.001**	+	5.96	**.015**	+	2.47	.116		4.28	**.039**	+
Specific root length (SRL)	2.59	.108		0.12	.726		0.07	.792		0.22	.639	
AMF colonization rate	0.03	.854		0.39	.530		<0.01	.931		0.08	.781	
Soil properties												
PC1 (C_org_, N, P → pH)	10.07	**.002**	−	1.76	.184		1.25	.263		3.01	*.083*	−
PC2 (soil K → pH)	1.31	.252		2.65	.103		0.46	.500		2.55	.110	
Soil organic carbon con.	10.43	**.001**	+	1.91	.168		1.24	.266		2.45	.117	
Soil N concentration	2.58	.108		0.20	.655		0.28	.594		0.83	.362	
Soil P concentration	1.61	.204		0.23	.632		0.38	.539		0.31	.579	
Soil K concentration	0.02	.899		0.40	.528		0.15	.703		0.72	.397	
Soil pH	11.25	**<.001**	−	8.29	**.004**	−	1.65	.199		7.17	**.007**	−

*Note*: Shown are Chi^2^ and *p*‐values (*p*). Significant effects (*p* < .05) are given in bold and marginally significant effects (*p* < .10) in italics. The plus‐icon behind *p*‐values indicates a positive relationship, while a minus‐icon indicates a negative relationship. Note that degrees of freedom (DF) were one for all variables fitted in the models.

**FIGURE 4:**
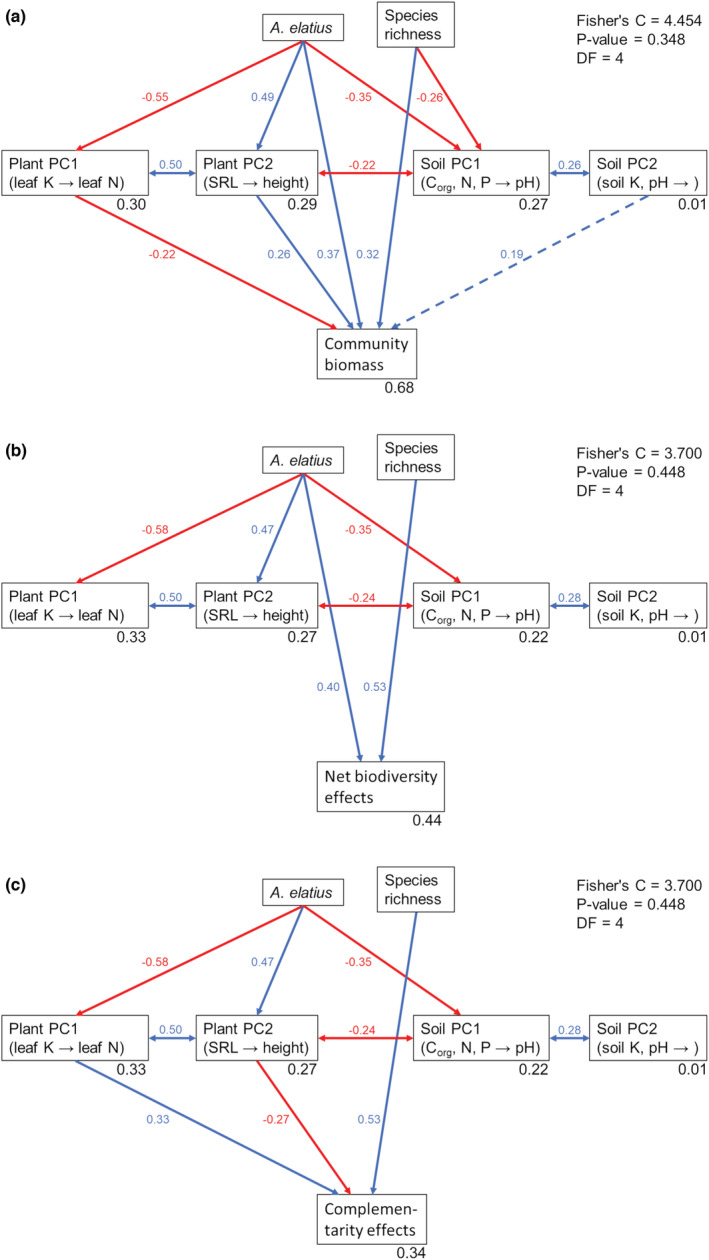
Piecewise structural equation models (SEM) exploring the effect of sown plant species richness, presence of the dominant species *A. elatius*, as well as plant and soil variables condensed as scores [PC1 and PC2] derived from principal component analysis on community biomass production (a), net biodiversity effects (b), and complementarity effects (c). Shown are Fisher's C, *p*‐values and degrees of freedom (DF) for each model. Solid arrows represent significant unidirectional relationships among variables (*p* < .05), dashed arrows represent marginal significant relationships (.05 < *p* < .1); blue arrows indicate positive relationships, and red arrows indicate negative relationships. Double‐headed arrows show correlated errors. Standardized parameter estimates are given next to the arrows. Marginal *R* ^2^ (based on fixed effects only) for component models with significant relationships is shown below the respective response variable.

Piecewise SEMs revealed that plant species richness and the presence of *A. elatius* negatively influenced soil PC1, i.e., increase in organic carbon, N and P, and decrease in soil pH (Figure 4). Moreover, we found a negative influence of *A. elatius* on plant PC1 (increase in CWM of leaf K, RLD, and plant height, decrease in CWM of leaf N and AMF colonization), and a positive influence on plant PC2 (increase in plant height and leaf P, decrease in SRL). Plant PC1 and PC2, and soil PC1 and PC2 showed positively correlated errors among each other, probably because plant PC1 and PC2 had both high loadings for plant height, and soil PC1 and PC2 had high loadings for soil pH. Additionally, we found a negatively correlated error between plant PC2 and soil PC1, i.e., communities with high CWM of plant height and leaf P had higher soil organic C, N, and P concentrations, while communities with high SRL had high soil pH. Finally, SEM showed a positive influence of plant species richness, the presence of *A. elatius*, and plant PC2, as well as a negative influence of plant PC1 on community biomass production explaining 68% of the variation (Figure 4a). For NEs, plant species richness and the presence of *A. elatius* had positive effects (44% explained variation, Figure 4b), and for CE, plant species richness and plant PC1 had positive effects and plant PC2 a negative effect (34% explained variation; Figure 4c). Although the presence of *A. elatius* and plant PC1 had significant effects on SEs, when considered as single predictors, we did not detect any influence of the PCs, plant species richness, or the presence of *A. elatius* in our SEM. Calculating a SEM without PCs revealed a direct positive effect of *A. elatius* and no effect of species richness on SEs, similar to the LMM results (data not shown).

Variance partitioning for productivity‐related variables indicated that, in case of community biomass production, most proportion (19%) is explained by plant traits; however, a nearly equal proportion is explained by the combination of plant traits, soil properties, and species richness (16%; Figure S5a). For NEs and CEs, plant species richness explained 27% of variation, respectively (Figure S5b,d). Furthermore, for NEs, soil properties explained 9% and all three predictors together 5% of variation (Figure S5b). For SEs, plant traits explained 6% and all other variables explained <5% of variation in biodiversity effects (Figure S5b–d).

## 4 DISCUSSION

### 4.1 Plant species richness and species identity influence plant community biomass production and biodiversity effects (H1)

Community biomass production and net biodiversity effects increased with plant species richness indicating that plant diversity is an important driver to maintain community productivity, which is in line with numerous previous studies (Cardinale et al., 2007; Marquard et al., 2009; Meyer et al., 2016; Tilman et al., 2001). This positive relationship can be explained by complementarity and selection effects. Both effects were also shown to drive positive plant diversity–productivity relationships in previous studies (Cardinale et al., 2007; Fargione et al., 2007; Marquard et al., 2009; Reich et al., 2012; Roscher et al., 2007); however, such studies have often shown that selection effects were smaller (or became smaller over time) than complementarity effects. In our study, selection and complementarity effects were about the same effect size after 15 years, which may be due to the fact that we had *A. elatius* in our species pool. The grass species *A. elatius* dominated the plant communities of our experiment since the beginning (Clark et al., 2020; Roscher et al., 2007), was the most productive species in monoculture and reached high biomass in plant mixtures. This was also found in other biodiversity experiments with a similar small species pool containing *A. elatius* (Roscher et al., 2016; Siebenkäs et al., 2016). Species like *A. elatius* are often dominant in the “target” community of our grasslands in the “real world” and have a significant impact on the ecosystem, which makes our study more realistic (Schmid et al., 2022). Despite the significant selection effects caused by *A. elatius*, we also found strong complementarity effects that increased with plant species richness and did not differ between communities with and without *A. elatius*. However, we detected that especially two species enhanced complementarity effects: *P. trivialis* and *T. pratense*. The grass species *P. trivialis* has a small growth stature (compared with other grass species in the Dominance Experiment), is, therefore, more adapted to shading and can contribute to the vertical niche filling, and thus increase the complementary use of light in the community (i.e., effectively use the light that reaches the lower herb layers; Lorentzen et al., 2008). By contrast, *T. pratense*, as a legume, may increase positive interactions due to facilitation (Roscher, Thein, et al., 2011). This indicates that not only species richness per se but also community composition play an important role in ecosystem functioning, which is also supported by previous work (Hooper & Dukes, 2004; Marquard et al., 2009).

### 4.2 Plant species richness and species identity influence community‐weighted means (CWM) of plant traits and AMF colonization rates (H2)

For two plant traits, plant height, and root length density, we found that their community means increased with plant species richness. For plant height, variance partitioning indicated that the increase was mainly caused by composition/abundance effects (explained 19% of the variation), i.e., the probability of tall‐growing species, such as *A. elatius*, being present and reaching high abundances in the community increased with plant species richness. However, the interactive effect of composition/abundance and adjustment effects explained a proportion of variance that was close to the same level (17%). This indicates that CWM of plant height was not only increased by the presence and high abundance of tall‐growing species but also by species growing taller in mixtures than in monoculture, probably to be able to compete for light with taller species and thus avoid extinction, as shown in previous studies testing biodiversity effects on plant trait variation (Lorentzen et al., 2008; Roscher, Schumacher, Gubsch, Lipowsky, Weigelt, Buchmann, Schulze, et al., 2018). As we do not have species‐specific data for root length density, we were not able to calculate variance partitioning for this trait.

Furthermore, for leaf nitrogen, leaf phosphorus and AMF colonization rates, and variance partitioning revealed an intriguing result: while composition/abundance effects increased with plant species richness, adjustment effects decreased. This result can explain why we found no change in CWM of these traits along the plant species richness gradient: the overall increase in CWM of leaf N, P, and AMF colonization due to the presence and larger abundances of *A. elatius* in mixtures (composition/abundance effect) were counterbalanced by generally decreasing values of these traits in other species (i.e., negative adjustment effects) with increasing plant species richness. A decrease in leaf nutrient concentrations with plant species richness was also shown in previous studies in the Jena Experiment (Abbas et al., 2013; Guiz et al., 2016, 2018). An explanation for this could be that subdominant smaller plant species change the allocation of resources, for example, plants in mixtures invest more resources (i.e., nutrients) into plant parts important for height growth (e.g., the stem) than, for example, into roots or leaves, which enables to grow taller and to compete with dominant species (Guiz et al., 2018). This could also be a reason for the decrease in the AMF colonization rate: plants in mixtures invest more resources into growth rather than maintain expensive mycorrhizal interactions. Another explanation for the decrease is a “dilution effect”: as plants produce more above‐ and belowground biomass in mixtures, leaf nutrient concentrations (and perhaps AMF colonization rates) are reduced in mixture plants compared with plants in monocultures (Guiz et al., 2018).

### 4.3 Plant species richness and species identity influence soil properties (H3)

As expected, concentrations of soil organic carbon increased with plant species richness and the presence of *A. elatius*, which is explainable by higher litter input (due to higher biomass production), higher quality and quantity of root exudates, and/or an increasing soil biota abundance (Eisenhauer et al., 2010; Fornara & Tilman, 2008; Lange et al., 2015). Also, total nitrogen increased with plant species richness and the presence of *A. elatius*. We did not measure concentrations of different nitrogen forms that are relevant for plant growth (especially nitrate and ammonium), but several studies have shown that inorganic N decreases along the plant species richness gradient (especially nitrate; Palmborg et al., 2005; Roscher et al., 2008). Thus, nitrogen forms that are not easily accessible to plants are likely to be responsible for the increase in total soil N, for example, nitrogen accumulated in soil organic matter or microbial biomass (Fornara & Tilman, 2008; Gubsch et al., 2011; Leimer et al., 2016).

Furthermore, we found that plant‐available phosphorus in soils increased with plant species richness; however, this was mainly explainable by the more frequent presence of dominant species in the mixtures of higher plant species richness (i.e., the grasses *A. elatius* and *D. glomerata*). One possible explanation for this finding is the change in soil pH, which coincides with the accumulation of soil organic matter with increasing plant species richness and the presence of these dominant grass species (Berendse et al., 1998). Soil pH is known as the “master soil variable”, because it influences many biological, chemical, and physical processes in the soil, including the composition of soil biota and the availability of soil nutrients, such as phosphorus for plants (Hinsinger et al., 2003; Neina, 2019). Optimum for P availability in soils are pH values between 6.0 and 7.5, while higher pH (pH > 7.5) increasingly limits P availability to plants due to the fixation by calcium (Clarkson & Hanson, 1980). The site of the Jena Experiment has generally high soil pH values (Roscher et al., 2004). In our study, we detected a decrease in soil pH from 7.40 ± 0.05 in monocultures to a pH of 7.32 ± 0.05 in 9‐species mixtures, which may appear small but can significantly affect the availability of phosphorus for plants and is supported by the negative correlation between soil pH and P in leaves and soil. On the contrary, plant‐available potassium in soils is less dependent on pH changes when soil pH is generally greater than 6.0, which is the case in the Jena Experiment. We also found no significant effect of plant species richness on plant‐available potassium and no correlation between leaf/soil potassium and soil pH. However, similar to plant‐available phosphorus, we found a positive influence of *A. elatius* on plant‐available potassium. This indicates that plant‐available potassium is mainly determined by the presence of dominant grasses. Grasses are known to accumulate more potassium in tissues than herbaceous plants (Schimmelpfennig et al., 2015; Tilman et al., 1999), and therefore their litter is higher in concentrations of potassium. In our experiment, the grass species *A. elatius* was the most productive species, and thus its potassium‐rich litter may result in more potassium being transferred to the soil, which can then explain the higher availability of soil K in plots with this species present.

Main processes that can lead to a change in soil pH are plant‐induced processes, such as the release of inorganic ions for uptake of nutrients, root exudation or respiration, and soil biota‐induced processes, such as biochemical transformations and decomposition of organic matter (Neina, 2019). Based on our data, it is not possible to disentangle which processes play the largest role in the decrease in soil pH with plant species richness and the presence of *A. elatius*. To understand this in more detail, further research is needed, but our results suggest that these changes in soil pH may influence plant growth via effects on the availability of nutrients.

### 4.4 Relationships between plant traits and soil properties

We found a negative correlation between soil pH and CWM of leaf phosphorus (and plant‐available phosphorus in soil), which supports the assumption that a decrease in soil pH from 7.4 to 7.3 lowers the P fixation and thus increases the availability for plants. Moreover, we found significant correlations between the community means of plant height and root length density and all soil properties (except plant‐available K). This suggests that communities with a high abundance of tall and exploitive species are able to positively influence their environment, i.e., decrease the soil pH, increase litter input, and promote the activity of soil biota (increase in soil organic carbon and total nitrogen), which leads to higher availability of soil nutrients, such as plant‐available phosphorus. We also found significant correlations between CWM of leaf nitrogen, phosphorus, and potassium, and plant‐available phosphorus and potassium in soils. As expected, the concentration of P and K in the leaves increased with higher concentrations of soil P and K—most likely, plants with higher P and K concentrations provide more plant‐available P and K in soils through litter input.

Principal component analysis including soil and plant variables shows an increase in CWM of plant height, soil organic carbon, total N, and plant‐available P with plant species richness, while community‐level SRL and soil pH decrease. The trajectory direction of the other variables can be explained by the proportion of grass species sown in the plots. Communities with grass species were characterized by high CWM of SLA, and leaf K, while communities without grass species had higher CWM of leaf N and AMF colonization rates. This is in line with previous studies, which showed that grasses are characterized by high leaf K concentrations but have low leaf N concentrations and were less connected to mycorrhizal fungi, compared with herbs and legumes (Eisenhauer et al., 2009; Schimmelpfennig et al., 2015; Tilman et al., 1999). In case of SLA, it was not a grass species effect per se that determined the CWM but rather an effect of our species selection and the nutrient uptake strategies of the species included in the experiment: most of the selected grass species are fast‐growing, and therefore have a high SLA, while the selected forbs are slow‐growing and therefore have a smaller SLA (Reich, 2014; Wright et al., 2004).

### 4.5 Direct and indirect effects of plant and soil variables on community biomass production and biodiversity effects (H4)

Mixed‐effects model analysis showed that community means of plant height, root length density, and leaf potassium concentration were the most important plant‐related drivers of biomass production and biodiversity effects. These results are mainly attributable to the presence of *A. elatius*, which increased community biomass production and CWM of plant height, leaf potassium, and community‐level RLD (via composition effects). At the same time, plant height and eventually RLD of the other species increased, when *A. elatius* was present in the community, in order to stay competitive for light and nutrients, which further increased community biomass production (via interactive impact of composition/abundance and adjustment effects). Similar positive effects of CWM of plant height on complementarity effects via dominant species were found in a recent study (Valencia et al., 2022).

In case of soil variables, the mixed‐effect model analysis revealed that soil organic carbon and soil pH had a strong influence on community biomass production and biodiversity effects (except SEs). As stated in the introduction, it is most likely that the accumulation of organic carbon and the lowering of soil pH mutually stimulated each other, increasing nutrient availability and thus overyielding.

Similar to the mixed‐effects model results, SEM showed direct effects of plant functional traits (plant PC1 and PC2) on community biomass production and complementarity effects; however, there was no significant influence of soil variables (soil PC1 and PC2) on productivity or biodiversity effects. This indicates that only plant functional traits have a direct influence, while the effect of soil characteristics (for example the effect of soil pH) must be indirect. One indirect way that the SEM suggests is that plant species richness and the presence of *A. elatius* alter soil characteristics, which in turn affects the expression of functional traits, thus influencing community biomass production and biodiversity effects (which is also supported by the results of the variance partitioning for the productivity‐related variables). A concrete mechanism for how species richness and dominant species increase biomass production based on our findings could be the following: high species richness and the presence of *A. elatius*, as the dominant species, decrease the soil pH over time (e.g., through root exudates and/or the accumulation of organic carbon). As a result, more nutrients are accessible for plants and soil biota. The higher nutrient availability, in turn, has a positive effect on the plants, i.e., they can invest more resources in height and root growth. This mainly favors plant species with tall growth and dense root systems resulting in an increase in biomass production in species‐rich communities. The increased biomass production then leads to greater litter input, which further increases microbial activity and thus decomposition (i.e., increased soil organic carbon and nitrogen) and further lowers soil pH, which strengthens the mechanism (i.e., feedback processes).

Finally, we want to point out that our investigations cannot fully disentangle whether it was the change in soil properties that changed the plant traits in the first place, or whether it was the other way round—the change in plant traits altered the soil characteristics. For this, one would need separate experiments that investigate this issue in a more controlled way, e.g., in greenhouse studies, where plant diversity effects and resulting nutrient dynamics are tested under different soil conditions, including soil pH and nutrient availability. This could be achieved, e.g., by liming or fertilizing the soil. Nevertheless, the present results suggest that soil properties and their link to plant functional traits, i.e., plant–soil interactions, should be considered in order to fully understand the mechanisms driving positive plant diversity–productivity relationships.

## AUTHOR CONTRIBUTIONS

**Peter Dietrich:** Conceptualization (equal); data curation (lead); formal analysis (lead); writing – original draft (lead). **Nico Eisenhauer:** Funding acquisition (equal); project administration (equal); supervision (supporting); writing – review and editing (equal). **Christiane Roscher:** Conceptualization (equal); data curation (supporting); formal analysis (supporting); funding acquisition (equal); project administration (equal); writing – original draft (supporting); writing – review and editing (equal).

## CONFLICT OF INTEREST STATEMENT

The authors declare no conflict of interest.

### OPEN RESEARCH BADGES

This article has earned an Open Data badge for making publicly available the digitally‐shareable data necessary to reproduce the reported results. The data is available at https://doi.org/10.25829/BWH0‐9W18.

## Supporting information


Appendix S1

## Data Availability

The data used in this study (Dietrich & Roscher, 2023) are publicly available in the Jena Experiment data portal (https://jexis.idiv.de/): https://doi.org/10.25829/BWH0‐9W18.
