# Supplementary material for: Linking plant diversity–productivity relationships to plant functional traits of dominant species and changes in soil properties in 15‐year‐old experimental grasslands
Source: Ecol Evol. 2023 Mar 8;13(3):e9883. doi: 10.1002/ece3.9883 (PMC9994614; doi:10.1002/ece3.9883)
Supplement: Supplementary file 1 — Appendix S1 [file ECE3-13-e9883-s001.docx]

**Appendix**

Journal: Ecology and Evolution

Title: Linking plant diversity–productivity relationships to plant functional traits of dominant species and changes in soil properties in 15-year old experimental grasslands

Authors: Peter Dietrich, Nico Eisenhauer, and Christiane Roscher

**Appendix Tables**

**Table S1** Summary of the two-species communities used for soil and plant sampling. Numbers in rows indicate whether we used both replicates (2), one of the two replicates (1), or none of the two (0). The last column indicates how often each plant species was present in the selected two-species plots.

|  | Arr ela | Alo pra | Dac glo | Poa tri | Ger pra | Tri pra | Tri rep | Ant syl | Phl pra | Total |
| --- | --- | --- | --- | --- | --- | --- | --- | --- | --- | --- |
| *Arrhenatherum elatius* (Arr ela) | - | 2 | 2 | 2 | 2 | 1 | 1 | 1 | 1 | 12 |
| *Alopecurus pratensis* (Alo pra) | 2 | - | 2 | 2 | 2 | 0 | 1 | 1 | 1 | 11 |
| *Dactylis glomerata* (Dac glo) | 2 | 2 | - | 2 | 2 | 1 | 1 | 1 | 1 | 12 |
| *Poa trivialis* (Poa tri) | 2 | 2 | 2 | - | 2 | 2 | 2 | 1 | 1 | 14 |
| *Geranium pratense* (Ger pra) | 2 | 2 | 2 | 2 | - | 2 | 2 | 0 | 0 | 12 |
| *Trifolium pratense* (Tri pra) | 1 | 0 | 1 | 2 | 2 | - | 1 | 2 | 1 | 10 |
| *Trifolium repens* (Tri rep) | 1 | 1 | 1 | 2 | 2 | 1 | - | 0 | 1 | 9 |

**Table S2** Summary of mixed-effect model analyses testing the effects of plant species richness (Sr) and presence of plant species (plant species ID) on productivity variables. Shown are Chi^2^ and P-values (P) for model 1 (upper part of the table) and model 2 (lower part of the table). For model 1, we fitted sown plant species richness first and then plant species ID, and for model 2, we fitted first plant species ID followed by sown plant species richness (for each plant species in separate models). Moreover, we fitted separate models with realized plant species richness as fixed effect, but without species ID. Significant effects (P < 0.05) are given in bold and marginally significant effects (P < 0.10) in italics. The plus-icon behind P-values indicates a significant increase, while a minus-icon indicates a decrease of the variable with species richness or presence of specific plant species. Note that degrees of freedom (DF) was one for all variables fitted in the models. Clarifications for species name abbreviations can be found in Appendix Table S1.

|  | Biomass production | | | Net biodiversity effects | | | Selection effects | | | Complementarity effects | | |
| --- | --- | --- | --- | --- | --- | --- | --- | --- | --- | --- | --- | --- |
| Model 1: | Chi^2^ | P |  | Chi^2^ | P |  | Chi^2^ | P |  | Chi^2^ | P |  |
| Species richness | 24.54 | **<0.001** | + | 25.23 | **<0.001** | + | 5.11 | **0.024** | + | 21.16 | **<0.001** | + |
| Realized Sr | 25.47 | **<0.001** | + | 15.13 | **<0.001** | + | 2.47 | 0.116 |  | 19.86 | **<0.001** | + |
| Sr + Alo pra | 1.54 | 0.215 |  | 3.04 | *0.081* | - | 0.98 | 0.322 |  | 1.90 | 0.168 |  |
| Sr + Arr ela | 42.48 | **<0.001** | + | 4.77 | **0.029** | + | 7.48 | **0.006** | + | 0.04 | 0.842 |  |
| Sr + Dac glo | 1.68 | 0.195 |  | 2.56 | 0.110 |  | <0.01 | 0.976 |  | 0.87 | 0.352 |  |
| Sr + Poa tri | 1.40 | 0.238 |  | 0.05 | 0.823 |  | 3.62 | *0.057* | - | 4.16 | **0.041** | + |
| Sr + Ger pra | 0.15 | 0.700 |  | 2.96 | *0.085* | - | 2.54 | 0.111 |  | 0.21 | 0.646 |  |
| Sr + Tri pra | 0.07 | 0.785 |  | 2.09 | 0.148 |  | 0.27 | 0.606 |  | 3.16 | *0.075* | + |
| Sr + Tri rep | 4.51 | **0.034** | + | 0.52 | 0.471 |  | 0.10 | 0.756 |  | 0.13 | 0.717 |  |
|  | Biomass production | | | Net biodiversity effects | | | Selection effects | | | Complementarity effects | | |
| Model 2: | Chi^2^ | P |  | Chi^2^ | P |  | Chi^2^ | P |  | Chi^2^ | P |  |
| Alo pra | 9.17 | **0.002** | + | 0.838 | 0.360 |  | 0.05 | 0.825 |  | 0.94 | 0.332 |  |
| + Sr | 16.91 | **<0.001** | + | 27.43 | **<0.001** | + | 6.05 | **0.014** | + | 22.12 | **<0.001** | + |
| Arr ela | 50.17 | **<0.001** | + | 11.16 | **<0.001** | + | 11.30 | **<0.001** | + | 4.61 | 0.032 |  |
| + Sr | 16.85 | **<0.001** | + | 18.84 | **<0.001** | + | 1.30 | 0.255 |  | 16.59 | **<0.001** | + |
| Dac glo | 1.00 | 0.316 |  | 0.61 | 0.433 |  | 0.96 | 0.326 |  | 1.58 | 0.209 |  |
| + Sr | 25.21 | **<0.001** | + | 27.17 | **<0.001** | + | 4.15 | **0.042** | + | 20.45 | **<0.001** | + |
| Poa tri | 1.09 | 0.300 |  | 3.45 | *0.063* | + | 0.41 | 0.521 |  | 12.18 | **<0.001** | + |
| + Sr | 24.84 | **<0.001** | + | 21.83 | **<0.001** | + | 8.33 | **0.004** | + | 13.14 | **<0.001** | + |
| Ger pra | 3.75 | *0.053* | + | 0.94 | 0.331 |  | 0.09 | 0.767 |  | 2.74 | *0.098* | + |
| + Sr | 20.94 | **<0.001** | + | 27.24 | **<0.001** | + | 7.56 | **0.006** | + | 18.64 | **<0.001** | + |
| Tri pra | 3.77 | *0.052* | + | 9.80 | **0.002** | + | 0.44 | 0.507 |  | 13.06 | **<0.001** | + |
| + Sr | 20.84 | **<0.001** | + | 17.52 | **<0.001** | + | 4.94 | **0.026** | + | 11.26 | **<0.001** | + |
| Tri rep | 0.50 | 0.480 |  | 8.02 | **0.005** | + | 2.00 | 0.158 |  | 4.00 | **0.046** | + |
| + Sr | 28.55 | **<0.001** | + | 17.72 | **<0.001** | + | 3.22 | *0.073* | + | 17.30 | **<0.001** | + |

**Table S3** Summary of mixed-effect model analyses testing the effects of plant species richness (Sr) and presence of plant species (plant species ID) on aboveground plant variables. Shown are Chi^2^ and P-values (P) for model 1 (upper part of the table) and model 2 (lower part of the table). For model 1, we fitted sown plant species richness first and then plant species ID, and for model 2, we fitted first plant species ID followed by sown plant species richness (for each plant species in separate models). Moreover, we fitted separate models with realized plant species richness as fixed effect, but without species ID. Significant effects (P < 0.05) are given in bold and marginally significant effects (P < 0.10) in italics. The plus-icon behind P-values indicates a significant increase, while a minus-icon indicates a decrease of the variable with species richness or presence of specific plant species. Note that degrees of freedom (DF) was one for all variables fitted in the models. Clarifications for species name abbreviations can be found in Appendix Table S1.

|  | Plant height | | | Specific leaf area | | | Leaf N concentration | | | Leaf P concentration | | | Leaf K concentration | | |
| --- | --- | --- | --- | --- | --- | --- | --- | --- | --- | --- | --- | --- | --- | --- | --- |
| Model 1: | Chi^2^ | P |  | Chi^2^ | P |  | Chi^2^ | P |  | Chi^2^ | P |  | Chi^2^ | P |  |
| Species richness | 13.07 | **<0.001** | + | 1.14 | 0.286 |  | 0.33 | 0.564 |  | 0.58 | 0.446 |  | 0.82 | 0.364 |  |
| Realized SR | 15.98 | **<0.001** | + | 0.56 | 0.454 |  | 0.34 | 0.561 |  | 0.31 | 0.576 |  | 1.11 | 0.293 |  |
| Sr + Alo pra | 3.67 | *0.055* | + | 3.24 | *0.072* | - | 0.26 | 0.107 |  | 0.11 | 0.915 |  | 0.43 | 0.512 |  |
| Sr + Arr ela | 71.42 | **<0.001** | + | 6.96 | **0.008** | + | 1.86 | 0.173 |  | 8.62 | **0.003** | + | 13.81 | **<0.001** | + |
| Sr + Dac glo | 0.64 | 0.425 |  | 0.04 | 0.832 |  | 0.31 | 0.575 |  | 0.62 | 0.431 |  | 3.42 | *0.065* | + |
| Sr + Poa tri | 4.96 | **0.026** | - | 6.85 | **0.009** | + | 3.45 | *0.063* | - | 8.49 | **0.004** | - | 0.68 | 0.411 |  |
| Sr + Ger pra | 0.36 | 0.546 |  | 7.27 | **0.007** | - | 0.02 | 0.901 |  | 3.20 | *0.073* | + | 2.86 | *0.091* |  |
| Sr + Tri pra | 1.27 | 0.259 |  | 0.93 | 0.335 |  | 11.53 | **<0.001** | + | 2.56 | 0.109 |  | 10.78 | **0.001** | - |
| Sr + Tri rep | 1.80 | 0.180 |  | 0.20 | 0.658 |  | 2.08 | 0.149 |  | 0.14 | 0.711 |  | 3.05 | *0.081* |  |
| Sr + Phl pra | 0.02 | 0.900 |  | 0.07 | 0.786 |  | 1.62 | 0.203 |  | 0.70 | 0.403 |  | 0.35 | 0.557 |  |
| Sr + Ant syl | 0.36 | 0.548 |  | 1.10 | 0.294 |  | <0.01 | 0.975 |  | 0.04 | 0.833 |  | <0.01 | 0.921 |  |
|  | Plant height | | | Specific leaf area | | | Leaf N concentration | | | Leaf P concentration | | | Leaf K concentration | | |
| Model 2: | Chi^2^ | P |  | Chi^2^ | P |  | Chi^2^ | P |  | Chi^2^ | P |  | Chi^2^ | P |  |
| Alo pra | 3.67 | *0.055* | + | 3.24 | *0.072* | - | 2.85 | *0.091* | - | 0.09 | 0.766 |  | 1.00 | 0.318 |  |
| + Sr | 9.57 | **0.002** | + | 5.20 | **0.023** | + | 0.08 | 0.775 |  | 0.50 | 0.478 |  | 0.26 | 0.613 |  |
| Arr ela | 78.98 | **<0.001** | + | 8.08 | **0.004** | + | 2.17 | 0.140 |  | 8.56 | **0.003** | + | 13.99 | **<0.001** | + |
| + Sr | 5.51 | **0.019** | + | 0.02 | 0.898 |  | 0.02 | 0.889 |  | 0.64 | 0.423 |  | 0.65 | 0.421 |  |
| Dac glo | 0.72 | 0.397 |  | 0.44 | 0.508 |  | 0.59 | 0.444 |  | 1.14 | 0.285 |  | 4.24 | **0.039** | + |
| + Sr | 12.99 | **<0.001** | + | 0.75 | 0.388 |  | 0.06 | 0.804 |  | 0.06 | 0.811 |  | <0.01 | 0.971 |  |
| Poa tri | 0.07 | 0.798 |  | 7.97 | **0.004** | + | 3.69 | *0.055* | - | 4.69 | **0.030** | - | 1.28 | 0.258 |  |
| + Sr | 17.96 | **<0.001** | + | 0.01 | 0.920 |  | 0.09 | 0.758 |  | 4.39 | **0.036** | + | 0.22 | 0.639 |  |
| Ger pra | 1.41 | 0.235 |  | 3.04 | *0.081* | - | 0.15 | 0.698 |  | 3.71 | *0.054* | + | 1.04 | 0.307 |  |
| + Sr | 12.02 | **<0.001** | + | 5.37 | **0.020** | + | 0.20 | 0.657 |  | 0.07 | 0.786 |  | 2.64 | 0.104 |  |
| Tri pra | 0.50 | 0.479 |  | 0.10 | 0.749 |  | 5.85 | **0.016** | + | 0.86 | 0.354 |  | 5.43 | **0.020** | - |
| + Sr | 13.84 | **<0.001** | + | 1.97 | 0.161 |  | 6.01 | **0.014** | - | 2.29 | 0.131 |  | 6.17 | **0.013** | + |
| Tri rep | 0.31 | 0.580 |  | 0.02 | 0.894 |  | 0.86 | 0.353 |  | 0.01 | 0.919 |  | 1.14 | 0.285 |  |
| + Sr | 14.56 | **<0.001** | + | 1.32 | 0.251 |  | 1.55 | 0.213 |  | 0.71 | 0.400 |  | 2.73 | *0.098* | + |

**Table S4** Summary of mixed-effect model analyses testing the effects of plant species richness (Sr) and presence of plant species (plant species ID) on root length density (RLD), specific root length (SRL), and AMF colonization rate. Shown are Chi^2^ and P-values (P) for model 1 (upper part of the table) and model 2 (lower part of the table). For model 1, we fitted sown plant species richness first and then plant species ID, and for model 2, we fitted first plant species ID followed by sown plant species richness (for each plant species in separate models). Moreover, we fitted separate models with realized plant species richness as fixed effect, but without species ID. Significant effects (P < 0.05) are given in bold and marginally significant effects (P < 0.10) in italics. The plus-icon behind P-values indicates a significant increase, while a minus-icon indicates a decrease of the variable with species richness or presence of specific plant species. Note that degrees of freedom (DF) was one for all variables fitted in the models. Clarifications for species name abbreviations can be found in Appendix Table S1.

|  | RLD | | | SRL | | | AMF colonization rate | | |
| --- | --- | --- | --- | --- | --- | --- | --- | --- | --- |
| Model 1: | Chi^2^ | P |  | Chi^2^ | P |  | Chi^2^ | P |  |
| Species richness | 10.41 | **0.001** | + | 3.75 | *0.053* | - | 0.26 | 0.610 |  |
| Realized SR | 8.57 | **0.003** | + | 4.92 | **0.027** | - | 0.47 | 0.494 |  |
| Sr + Alo pra | 7.12 | **0.008** | + | 0.89 | 0.345 |  | 0.18 | 0.667 |  |
| Sr + Arr ela | 10.75 | **0.001** | + | 0.73 | 0.392 |  | 0.08 | 0.774 |  |
| Sr + Dac glo | 0.19 | 0.663 |  | 4.25 | **0.039** | + | 4.31 | **0.038** | - |
| Sr + Poa tri | 0.57 | 0.451 |  | 5.23 | **0.022** | + | 4.04 | **0.044** | - |
| Sr + Ger pra | 6.19 | **0.013** | - | 13.06 | **<0.001** | - | 2.11 | 0.147 |  |
| Sr + Tri pra | 5.99 | **0.014** | - | 0.82 | 0.366 |  | 4.77 | **0.029** | + |
| Sr + Tri rep | 1.20 | 0.273 |  | 1.17 | 0.279 |  | 1.12 | 0.290 |  |
| Sr + Phl pra | 0.05 | 0.816 |  | 0.02 | 0.888 |  | 0.50 | 0.481 |  |
| Sr + Ant syl | 0.17 | 0.677 |  | 1.10 | 0.295 |  | 0.13 | 0.719 |  |
|  | RLD | | | SRL | | | AMF colonization rate | | |
| Model 2: | Chi^2^ | P |  | Chi^2^ | P |  | Chi^2^ | P |  |
| Alo pra | 14.08 | **<0.001** | + | 3.31 | *0.069* | - | 0.41 | 0.523 |  |
| + Sr | 3.44 | *0.064* | + | 1.33 | 0.249 |  | 0.04 | 0.847 |  |
| Arr ela | 17.77 | **<0.001** | + | 2.95 | 0.086 |  | 0.26 | 0.608 |  |
| + Sr | 3.39 | *0.066* | + | 1.53 | 0.216 |  | 0.08 | 0.778 |  |
| Dac glo | 1.05 | 0.305 |  | 0.55 | 0.459 |  | 4.13 | **0.042** | - |
| + Sr | 9.54 | **0.002** | + | 7.45 | **0.006** | - | 0.44 | 0.506 |  |
| Poa tri | 3.98 | **0.046** | + | 1.08 | 0.299 |  | 4.00 | **0.045** | - |
| + Sr | 6.99 | **0.008** | + | 7.90 | **0.005** | - | 0.30 | 0.583 |  |
| Ger pra | 0.15 | 0.703 |  | 16.78 | **<0.001** | - | 0.92 | 0.339 |  |
| + Sr | 16.45 | **<0.001** | + | 0.03 | 0.872 |  | 1.45 | 0.228 |  |
| Tri pra | 0.14 | 0.707 |  | 3.28 | *0.070* | - | 2.24 | 0.134 |  |
| + Sr | 16.25 | **<0.001** | + | 1.28 | 0.257 |  | 2.79 | *0.095* | - |
| Tri rep | 0.33 | 0.563 |  | 0.04 | 0.840 |  | 0.31 | 0.577 |  |
| + Sr | 11.28 | **<0.001** | + | 4.88 | **0.027** | **-** | 1.07 | 0.301 |  |

**Table S5** Summary of mixed-effect model analyses testing the effects of plant species richness (Sr) and presence of plant species (plant species ID) on soil variables. Shown are Chi^2^ and P-values (P) for model 1 (upper part of the table) and model 2 (lower part of the table). For model 1, we fitted sown plant species richness first and then plant species ID, and for model 2, we fitted first plant species ID followed by sown plant species richness (for each plant species in separate models). Moreover, we fitted separate models with realized plant species richness as fixed effect, but without species ID. Significant effects (P < 0.05) are given in bold and marginally significant effects (P < 0.10) in italics. The plus-icon behind P-values indicates a significant increase, while a minus-icon indicates a decrease of the variable with species richness or presence of specific plant species. Note that degrees of freedom (DF) was one for all variables fitted in the models. Clarifications for species name abbreviations can be found in Appendix Table S1.

|  | Organic carbon con. | | | Soil N concentration | | | Soil P concentration | | | Soil K concentration | | | Soil pH | | |
| --- | --- | --- | --- | --- | --- | --- | --- | --- | --- | --- | --- | --- | --- | --- | --- |
| Model 1: | Chi^2^ | P |  | Chi^2^ | P |  | Chi^2^ | P |  | Chi^2^ | P |  | Chi^2^ | P |  |
| Species richness | 19.25 | **<0.001** | + | 11.61 | **<0.001** | + | 4.45 | **0.035** | + | 3.52 | *0.060* | + | 14.52 | **<0.001** | - |
| Realized SR | 16.27 | **<0.001** | + | 9.71 | **0.002** | + | 4.11 | **0.043** | + | 4.26 | **0.039** | + | 13.77 | **<0.001** | - |
| Sr + Alo pra | 0.02 | 0.889 |  | <0.01 | 0.949 |  | <0.01 | 0.957 |  | 0.98 | 0.322 |  | 0.98 | 0.321 |  |
| Sr + Arr ela | 8.32 | **0.004** | + | 4.39 | **0.036** | + | 9.31 | **0.002** | + | 5.18 | **0.023** | + | 9.61 | **0.002** | - |
| Sr + Dac glo | 0.02 | 0.898 |  | 0.03 | 0.870 |  | 5.11 | **0.024** | + | 0.53 | 0.468 |  | 1.98 | 0.159 |  |
| Sr + Poa tri | 1.35 | 0.244 |  | 2.24 | 0.135 |  | 0.29 | 0.591 |  | 0.96 | 0.326 |  | 0.02 | 0.900 |  |
| Sr + Ger pra | 0.05 | 0.825 |  | 0.02 | 0.879 |  | 0.14 | 0.712 |  | <0.01 | 0.928 |  | 0.50 | 0.479 |  |
| Sr + Tri pra | 0.28 | 0.599 |  | 0.16 | 0.688 |  | 4.92 | **0.027** | - | 5.05 | **0.025** | - | 4.55 | **0.033** | + |
| Sr + Tri rep | 0.31 | 0.579 |  | 1.51 | 0.220 |  | 0.23 | 0.629 |  | 0.44 | 0.507 |  | 0.09 | 0.762 |  |
| Sr + Phl pra | 0.23 | 0.633 |  | 0.12 | 0.734 |  | 1.13 | 0.288 |  | 3.63 | *0.057* |  | 0.71 | 0.399 |  |
| Sr + Ant syl | 1.58 | 0.209 |  | 0.93 | 0.336 |  | 0.66 | 0.416 |  | 0.59 | 0.442 |  | 1.87 | 0.171 |  |
|  | Organic carbon con. | | | Soil N concentration | | | Soil P concentration | | | Soil K concentration | | | Soil pH | | |
| Model 2: | Chi^2^ | P |  | Chi^2^ | P |  | Chi^2^ | P |  | Chi^2^ | P |  | Chi^2^ | P |  |
| Alo pra | 4.22 | **0.040** | + | 2.84 | *0.092* | + | 0.76 | 0.384 |  | 2.82 | *0.093* | + | 1.00 | 0.318 |  |
| + Sr | 15.05 | **<0.001** | + | 8.78 | **0.003** | + | 3.32 | *0.069* | + | 1.16 | 0.281 |  | 14.51 | **<0.001** | - |
| Arr ela | 17.71 | **<0.001** | + | 11.43 | **<0.001** | + | 12.38 | **<0.001** | + | 7.54 | **0.006** | + | 18.61 | **<0.001** | - |
| + Sr | 9.86 | **0.002** | + | 4.57 | **0.033** | + | 0.27 | 0.606 |  | 0.26 | 0.610 |  | 5.52 | **0.019** | - |
| Dac glo | 3.33 | *0.068* | + | 1.99 | 0.158 |  | 10.80 | **0.001** | + | 3.14 | *0.077* | + | 7.89 | **0.005** | - |
| + Sr | 15.94 | **<0.001** | + | 9.65 | **0.002** | + | 0.31 | 0.576 |  | 1.07 | 0.302 |  | 8.61 | **0.003** | - |
| Poa tri | 0.93 | 0.336 |  | 0.10 | 0.747 |  | 0.12 | 0.733 |  | 2.49 | 0.114 |  | 3.00 | *0.083* | - |
| + Sr | 19.68 | **<0.001** | + | 13.74 | **<0.001** | + | 4.37 | **0.037** | + | 1.41 | 0.235 |  | 11.54 | **<0.001** | - |
| Ger pra | 3.13 | *0.077* | + | 2.09 | 0.148 |  | 0.28 | 0.595 |  | 0.62 | 0.431 |  | 5.30 | **0.021** | - |
| + Sr | 16.17 | **<0.001** | + | 9.54 | **0.002** | + | 4.01 | **0.045** | + | 2.49 | 0.114 |  | 9.72 | **0.002** | - |
| Tri pra | 2.34 | 0.126 |  | 1.68 | 0.196 |  | 0.71 | 0.399 |  | 0.99 | 0.319 |  | 0.03 | 0.872 |  |
| + Sr | 17.19 | **<0.001** | + | 10.09 | **0.001** | + | 8.62 | **0.003** | + | 7.54 | **0.006** | + | 19.05 | **<0.001** | - |
| Tri rep | 6.03 | **0.014** | + | 7.65 | **0.006** | + | 0.33 | 0.568 |  | 0.11 | 0.740 |  | 2.52 | 0.113 |  |
| + Sr | 13.53 | **<0.001** | + | 5.47 | **0.019** | + | 4.04 | **0.045** | + | 3.53 | *0.060* | + | 12.10 | **<0.001** | - |

**Table S6** Summary of ANOVA results testing the effects of block, sown plant species richness, and mixture identity on composition effects, adjustment effects and CWM of plant height, specific leaf area (SLA), leaf nitrogen (N), phosphorus (P), and potassium (K) concentrations, and AMF colonization rates. Shown are degrees of freedom (DF), Chi^2^ and P-values (P). Significant effects (P < 0.05) are given in bold and marginally significant effects (P < 0.10) in italics. The plus-icon behind P-values indicates a significant increase, while a minus-icon indicates a decrease of the variable with species richness.

|  | Plant height | | | | SLA | | | | Leaf N concentrations | | | |
| --- | --- | --- | --- | --- | --- | --- | --- | --- | --- | --- | --- | --- |
|  | DF | F | P |  | DF | F | P |  | DF | F | P |  |
| Composition effect |  |  |  |  |  |  |  |  |  |  |  |  |
| Block | 3 | 5.24 | **0.004** |  | 3 | 0.25 | 0.859 |  | 3 | 1.72 | 0.183 |  |
| Species richness | 1 | 180.92 | **<0.001** | + | 1 | 4.32 | **0.046** | + | 1 | 12.74 | **0.001** | + |
| Mixture identity | 42 | 14.78 | **<0.001** |  | 42 | 2.04 | **0.021** |  | 42 | 5.58 | **<0.001** |  |
| Residuals | 31 |  |  |  | 31 |  |  |  | 31 |  |  |  |
| Adjustment effect |  |  |  |  |  |  |  |  |  |  |  |  |
| Block | 3 | 0.27 | 0.846 |  | 3 | 0.43 | 0.734 |  | 3 | 1.64 | 0.200 |  |
| Species richness | 1 | 17.81 | **<0.001** | + | 1 | 0.71 | 0.405 |  | 1 | 5.44 | **0.026** | - |
| Mixture identity | 42 | 1.06 | 0.440 |  | 42 | 1.29 | 0.232 |  | 42 | 1.73 | *0.057* |  |
| Residuals | 31 |  |  |  | 31 |  |  |  | 31 |  |  |  |
| CWM |  |  |  |  |  |  |  |  |  |  |  |  |
| Block | 3 | 4.11 | **0.015** |  | 3 | 1.62 | 0.205 |  | 3 | 1.91 | 0.148 |  |
| Species richness | 1 | 187.96 | **<0.001** | + | 1 | 15.98 | **<0.001** | + | 1 | 0.42 | 0.522 |  |
| Mixture identity | 42 | 5.87 | **<0.001** |  | 42 | 2.64 | 0.003 |  | 42 | 2.07 | **0.019** |  |
| Residuals | 31 |  |  |  | 31 |  |  |  | 31 |  |  |  |
|  | Leaf P concentrations | | | | Leaf K concentrations | | | | AMF colonization rates | | | |
|  | DF | F | P |  | DF | F | P |  | DF | F | P |  |
| Composition effect |  |  |  |  |  |  |  |  |  |  |  |  |
| Block | 3 | 1.61 | 0.207 |  | 3 | 0.90 | 0.450 |  | 3 | 1.45 | 0.246 |  |
| Species richness | 1 | 50.82 | **<0.001** | + | 1 | 11.46 | **0.002** | + | 1 | 10.70 | **0.003** | + |
| Mixture identity | 42 | 6.57 | **<0.001** |  | 42 | 4.10 | **<0.001** |  | 42 | 4.03 | **<0.001** |  |
| Residuals | 31 |  |  |  | 31 |  |  |  | 31 |  |  |  |
| Adjustment effect |  |  |  |  |  |  |  |  |  |  |  |  |
| Block | 3 | 1.87 | 0.156 |  | 3 | 3.28 | **0.034** |  | 3 | 2.57 | *0.072* |  |
| Species richness | 1 | 10.23 | **0.003** | - | 1 | 0.17 | 0.681 |  | 1 | 7.33 | **0.011** | - |
| Mixture identity | 42 | 2.50 | **0.005** |  | 42 | 3.21 | **<0.001** |  | 42 | 1.77 | *0.051* |  |
| Residuals | 31 |  |  |  | 31 |  |  |  | 31 |  |  |  |
| CWM |  |  |  |  |  |  |  |  |  |  |  |  |
| Block | 3 | 1.07 | 0.376 |  | 3 | 0.71 | 0.554 |  | 3 | 1.60 | 0.209 |  |
| Species richness | 1 | 2.35 | 0.136 |  | 1 | 15.12 | **<0.001** | + | 1 | 0.66 | 0.422 |  |
| Mixture identity | 42 | 2.24 | **0.011** |  | 42 | 5.30 | **<0.001** |  | 42 | 1.27 | 0.248 |  |
| Residuals | 31 |  |  |  | 31 |  |  |  | 31 |  |  |  |

**Table S7** Summary of variance partitioning results for plant height, specific leaf area (SLA), leaf nitrogen (N), phosphorus (P), and potassium (K) concentrations, and AMF colonization rates. Shown is explained variation by block, plant species richness (SR) and mixture identity (ID), remaining unexplained variation (error), in total, as well as partitioned into composition effects, adjustment effects, and interactive effects of composition and adjustment effects (comp. x adjust. effect).

|  | Plant height | | | | SLA | | | |
| --- | --- | --- | --- | --- | --- | --- | --- | --- |
|  | Composition effect | Adjustment effect | Comp. x adjust. effect | Total | Composition effect | Adjustment effect | Comp. x adjust. effect | Total |
| Block | 0.02 | <0.01 | 0.01 | 0.03 | 0.01 | 0.01 | 0.01 | 0.03 |
| SR | 0.19 | 0.04 | 0.17 | 0.39 | 0.05 | 0.01 | 0.04 | 0.10 |
| Mixture ID | 0.64 | 0.10 | -0.22 | 0.52 | 1.02 | 0.56 | -0.90 | 0.68 |
| Error | 0.03 | 0.07 | -0.03 | 0.06 | 0.37 | 0.32 | -0.50 | 0.19 |
|  | Leaf N concentrations | | | | Leaf P concentrations | | | |
|  | Composition effect | Adjustment effect | Comp. x adjust. effect | Total | Composition effect | Adjustment effect | Comp. x adjust. effect | Total |
| Block | 0.01 | 0.04 | <0.01 | 0.05 | 0.01 | 0.04 | -0.03 | 0.02 |
| SR | 0.02 | 0.04 | -0.06 | <0.01 | 0.16 | 0.07 | -0.21 | 0.02 |
| Mixture ID | 0.39 | 0.55 | -0.24 | 0.70 | 0.85 | 0.70 | -0.83 | 0.72 |
| Error | 0.05 | 0.24 | -0.04 | 0.25 | 0.10 | 0.21 | -0.06 | 0.24 |
|  | Leaf K concentrations | | | | AMF colonization rates | | | |
|  | Composition effect | Adjustment effect | Comp. x adjust. effect | Total | Composition effect | Adjustment effect | Comp. x adjust. effect | Total |
| Block | 0.01 | 0.02 | -0.03 | 0.01 | 0.01 | 0.05 | -0.01 | 0.05 |
| SR | 0.05 | <0.01 | 0.01 | 0.06 | 0.02 | 0.05 | -0.06 | 0.01 |
| Mixture ID | 0.70 | 0.31 | -0.20 | 0.82 | 0.29 | 0.50 | -0.19 | 0.59 |
| Error | 0.13 | 0.07 | -0.08 | 0.11 | 0.05 | 0.21 | 0.09 | 0.35 |

**Table S8** Correlation matrix for soil properties and plant traits. Shown are P-values and coefficients of correlation (r). Significant correlations (P < 0.05) are given in bold and marginally significant correlations (P < 0.10) in italics.

| P-values | | Soil properties | | | | Plant traits | | | | | | | |
| --- | --- | --- | --- | --- | --- | --- | --- | --- | --- | --- | --- | --- | --- |
|  |  | Org. C | Soil N | Soil P | Soil K | Height | SLA | Leaf N | Leaf P | Leaf K | RLD | SRL | AMF |
| Soil proper-ties | Soil pH | **0.001** | **0.001** | **<0.001** | 0.778 | **0.005** | 0.379 | 0.348 | **0.026** | 0.205 | **0.020** | 0.310 | 0.396 |
|  | Org. C |  | **<0.001** | **<0.001** | 0.442 | **0.002** | 0.877 | *0.072* | *0.052* | 0.154 | **0.004** | *0.086* | 0.367 |
|  | Soil N |  |  | **<0.001** | 0.732 | **0.015** | 0.989 | 0.151 | 0.112 | 0.311 | **0.011** | 0.111 | 0.483 |
|  | Soil P |  |  |  | 0.252 | **0.004** | 0.329 | **0.019** | **0.017** | **0.017** | **0.004** | 0.289 | *0.098* |
|  | Soil K |  |  |  |  | 0.262 | 0.761 | **0.035** | 0.313 | **0.041** | 0.359 | 0.270 | 0.429 |
| Plant traits | Height |  |  |  |  |  | 0.245 | **0.043** | **0.044** | **0.015** | **0.001** | *0.092* | 0.215 |
|  | SLA |  |  |  |  |  |  | 0.106 | 0.918 | **0.012** | 0.197 | 0.147 | **0.005** |
|  | Leaf N |  |  |  |  |  |  |  | 0.266 | **<0.001** | **0.005** | 0.747 | **<0.001** |
|  | Leaf P |  |  |  |  |  |  |  |  | 0.159 | 0.326 | *0.099* | 0.718 |
|  | Leaf K |  |  |  |  |  |  |  |  |  | **0.009** | 0.776 | **<0.001** |
|  | RLD |  |  |  |  |  |  |  |  |  |  | 0.573 | **0.030** |
|  | SRL |  |  |  |  |  |  |  |  |  |  |  | *0.068* |
| Coefficient of correlation (r) | | Soil properties | | | | Plant traits | | | | | | | |
|  |  | Org. C | Soil N | Soil P | Soil K | Height | SLA | Leaf N | Leaf P | Leaf K | RLD | SRL | AMF |
| Soil proper-ties | Soil pH | -0.44 | -0.40 | -0.53 | 0.23 | -0.37 | -0.14 | 0.02 | -0.30 | -0.12 | -0.26 | 0.13 | 0.11 |
|  | Org. C |  | 0.87 | 0.61 | 0.21 | 0.50 | 0.03 | -0.24 | 0.30 | 0.21 | 0.47 | -0.24 | -0.06 |
|  | Soil N |  |  | 0.57 | 0.05 | 0.33 | 0.01 | -0.17 | 0.19 | 0.13 | 0.39 | -0.22 | -0.03 |
|  | Soil P |  |  |  | 0.28 | 0.34 | 0.20 | -0.28 | 0.39 | 0.43 | 0.39 | -0.13 | -0.20 |
|  | Soil K |  |  |  |  | 0.20 | 0.10 | -0.28 | 0.20 | 0.43 | 0.15 | -0.20 | 0.02 |
| Plant traits | Height |  |  |  |  |  | 0.27 | -0.20 | 0.30 | 0.46 | 0.49 | -0.32 | -0.08 |
|  | SLA |  |  |  |  |  |  | -0.09 | -0.02 | 0.47 | 0.20 | 0.27 | -0.32 |
|  | Leaf N |  |  |  |  |  |  |  | -0.08 | -0.59 | -0.34 | -0.06 | 0.61 |
|  | Leaf P |  |  |  |  |  |  |  |  | 0.30 | 0.06 | -0.20 | 0.04 |
|  | Leaf K |  |  |  |  |  |  |  |  |  | 0.40 | 0.09 | -0.51 |
|  | RLD |  |  |  |  |  |  |  |  |  |  | -0.05 | -0.25 |
|  | SRL |  |  |  |  |  |  |  |  |  |  |  | -0.43 |

**Table S9** Correlation matrix for condensed soil and plant variables derived from principal component analyses. Shown are P-values.

| P-Values | Plant PC2 | Soil PC1 | Soil PC2 |
| --- | --- | --- | --- |
| Plant PC1 | 0.518 | 0.452 | 0.911 |
| Plant PC2 |  | 0.154 | 0.634 |
| Soil PC1 |  |  | 0.813 |

**Appendix sections**

**Section S1**

Relationships between plant traits and soil properties (correlation matrix results)

We found several significant correlations among plant functional traits, but the correlation coefficients were small in most cases (r < 0.50; Appendix Table S8). Significant correlations with a r > 0.50 were found between CWMs of leaf K and leaf N (negative), between CWMs of AMF colonization rates and leaf N (positive), and between CWMs of AMF colonization rates and leaf K (negative; Appendix Table S8). For soil properties, almost all soil variables were significantly correlated with each other, with the exception of plant-available K, which showed no correlation with other soil properties (Appendix Table S8). Soil organic carbon, total nitrogen, and plant-available phosphorus were positively correlated with each other, and negatively correlated with soil pH (with correlation coefficients between 0.40 and 0.87; Appendix Table S8). Almost all CWMs of plant traits showed significant correlations with soil properties, except SLA, SRL, and AMF colonization rates (no significant correlations with soil properties; Appendix Table S8). CWMs of plant height and RLD were positively correlated with soil organic C, total N, and plant-available P, and negatively correlated with soil pH. CWM of leaf N was negatively and CWM of leaf K was positively correlated with plant-available P and K in soil. CWM of leaf P was negatively correlated with soil pH, and positively with plant-available P. Most of these relationships showed low correlation coefficients between 0.26 and 0.50 (Appendix Table S8). There were no significant correlations between plant PC1 and PC2, and soil PC1 and PC2 (Appendix Table S9).

**Appendix figures**

**Figure S1** Null model for structural equation modelling to test the effects of plant species richness, presence of *A. elatius*, and plant and soil variables condensed as PC scores derived from principle component analyses on community biomass production, net biodiversity effects (NE), selection effects (SE) and complementarity effects (CE), respectively.

**
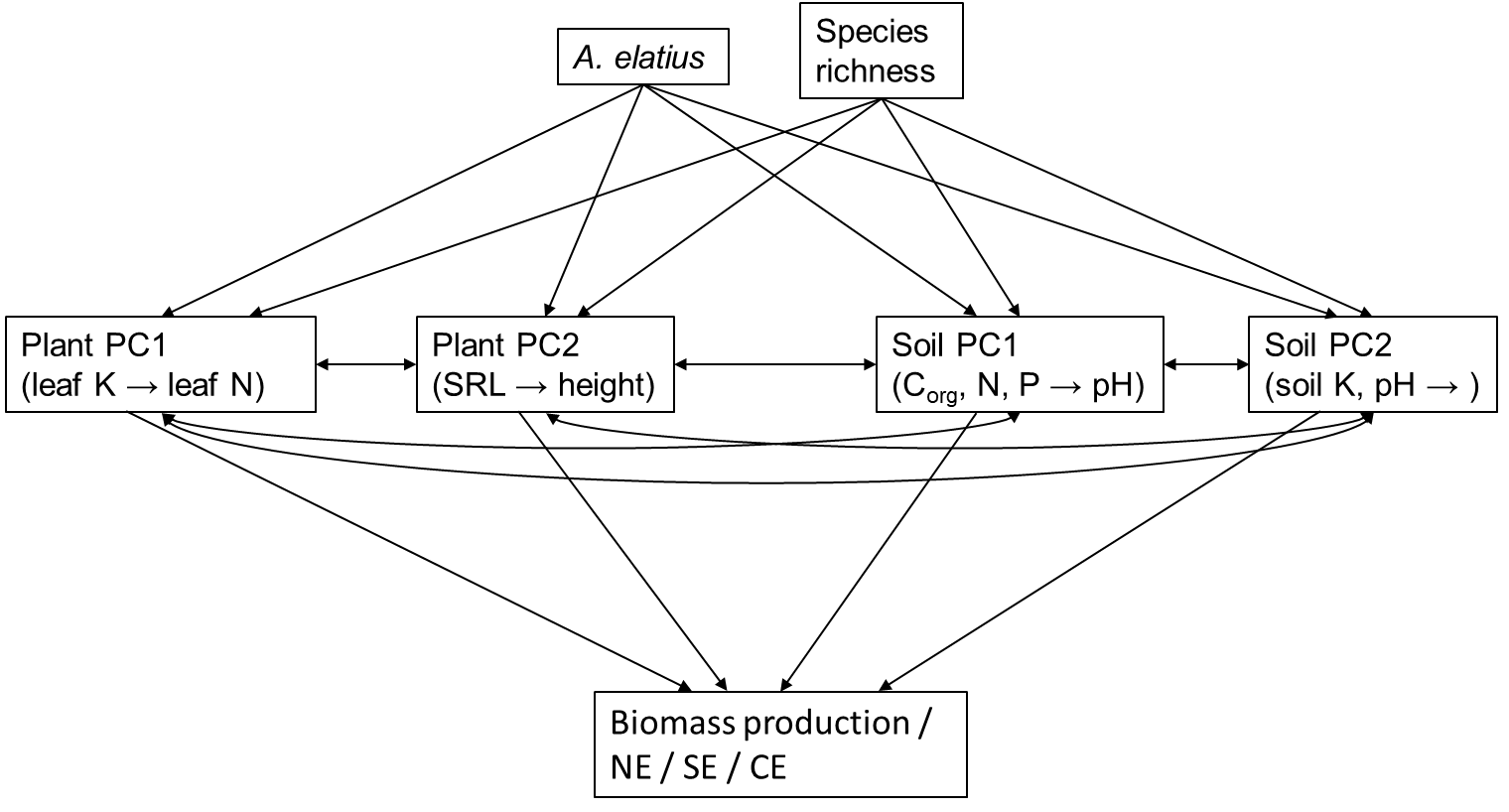
**

**Figure S2** Relationship between sown plant species richness (i.e., number of plant species sown in 2002) and realized plant species richness (i.e., number of plant species found in the plots in 2017).

**
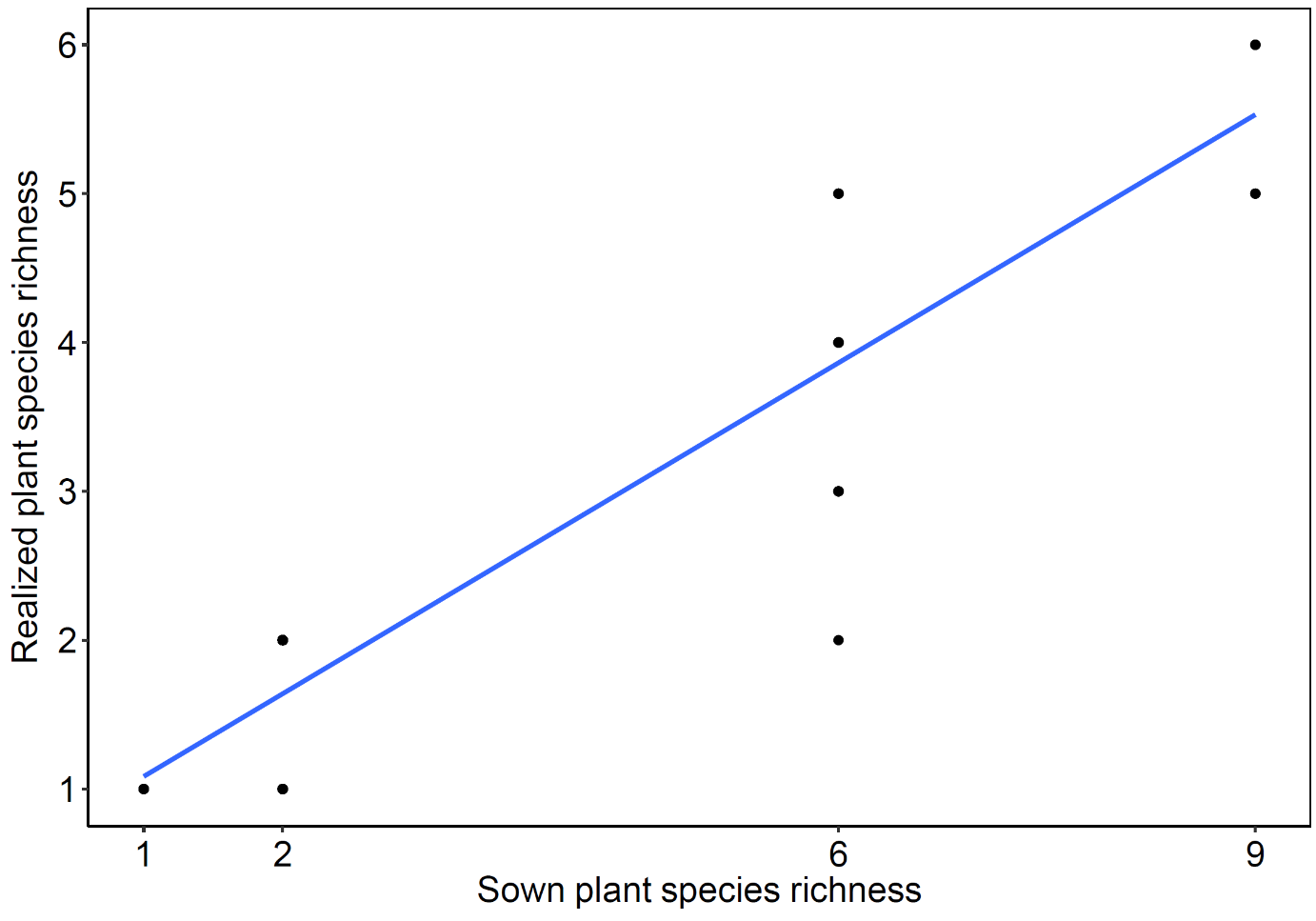
**

**Figure S3** Standardized principal components analysis (“plant PCA”; first vs. second axes) of 84 plant communities characterized by eight plant variables (community-level root length density [RLD] and specific root length [SRL], and CWM of plant height [Height], specific leaf area [SLA], leaf nitrogen [Leaf.N], leaf phosphorus [Leaf.P] and leaf potassium [Leaf.K] concentrations, and AMF colonization rates [AMF]). Shown are sown plant species richness groups as ellipses indicating the standard deviation of point scores for each group (1, 2, 6, and 9 plant species). Different colors indicate the affiliation to plant species richness groups.

**
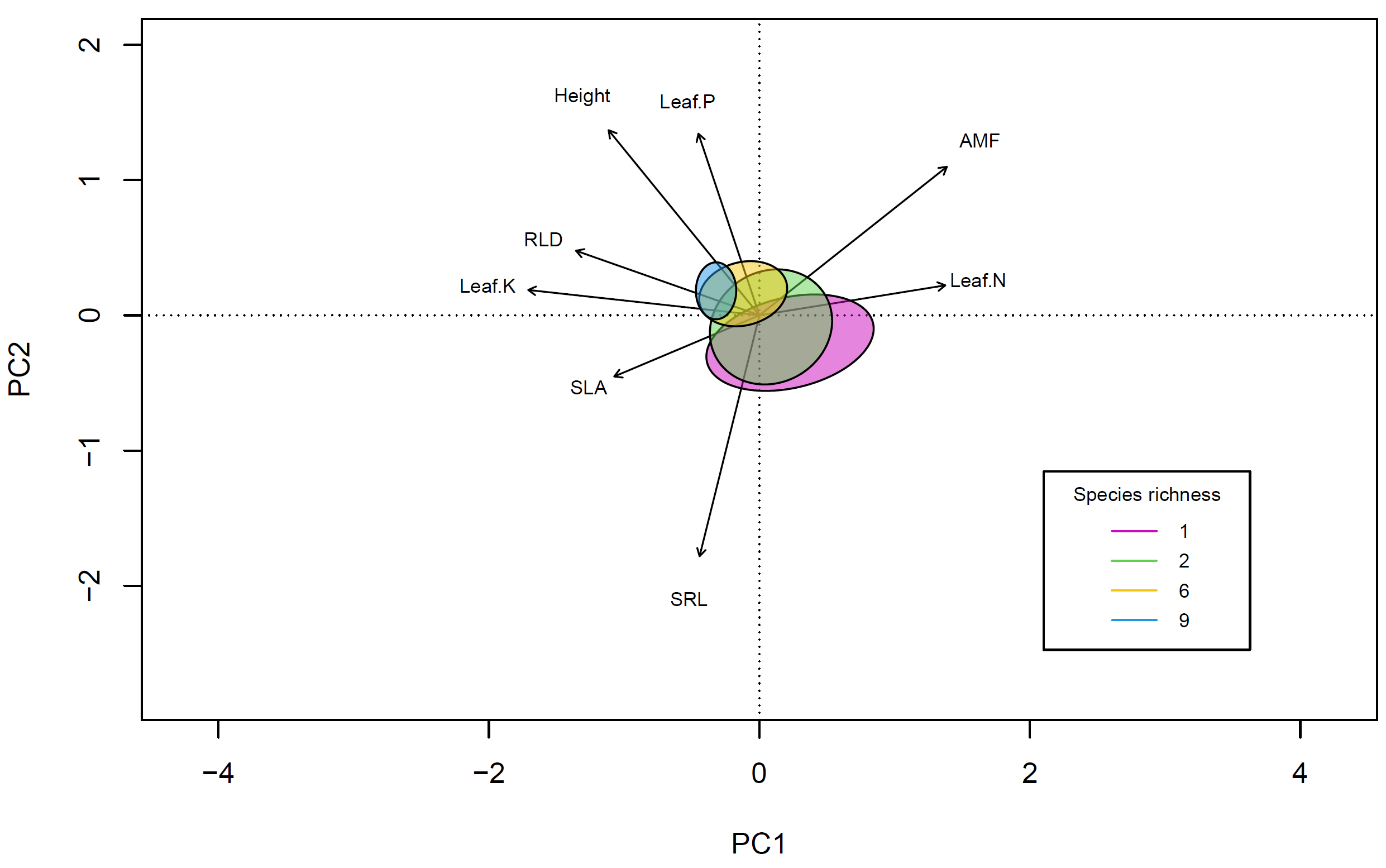
**

**Figure S4** Standardized principal components analysis (“soil PCA”; first vs. second axes) of 85 plant communities characterized by five soil variables (concentrations of organic carbon [Corg], total nitrogen [Soil.N], plant-available phosphorus [Soil.P] and plant-available potassium [Soil.K], as well as soil pH). Shown are sown plant species richness groups as ellipses indicating the standard deviation of point scores for each group (1, 2, 6, and 9 plant species). Different colors indicate the affiliation to the groups.

**
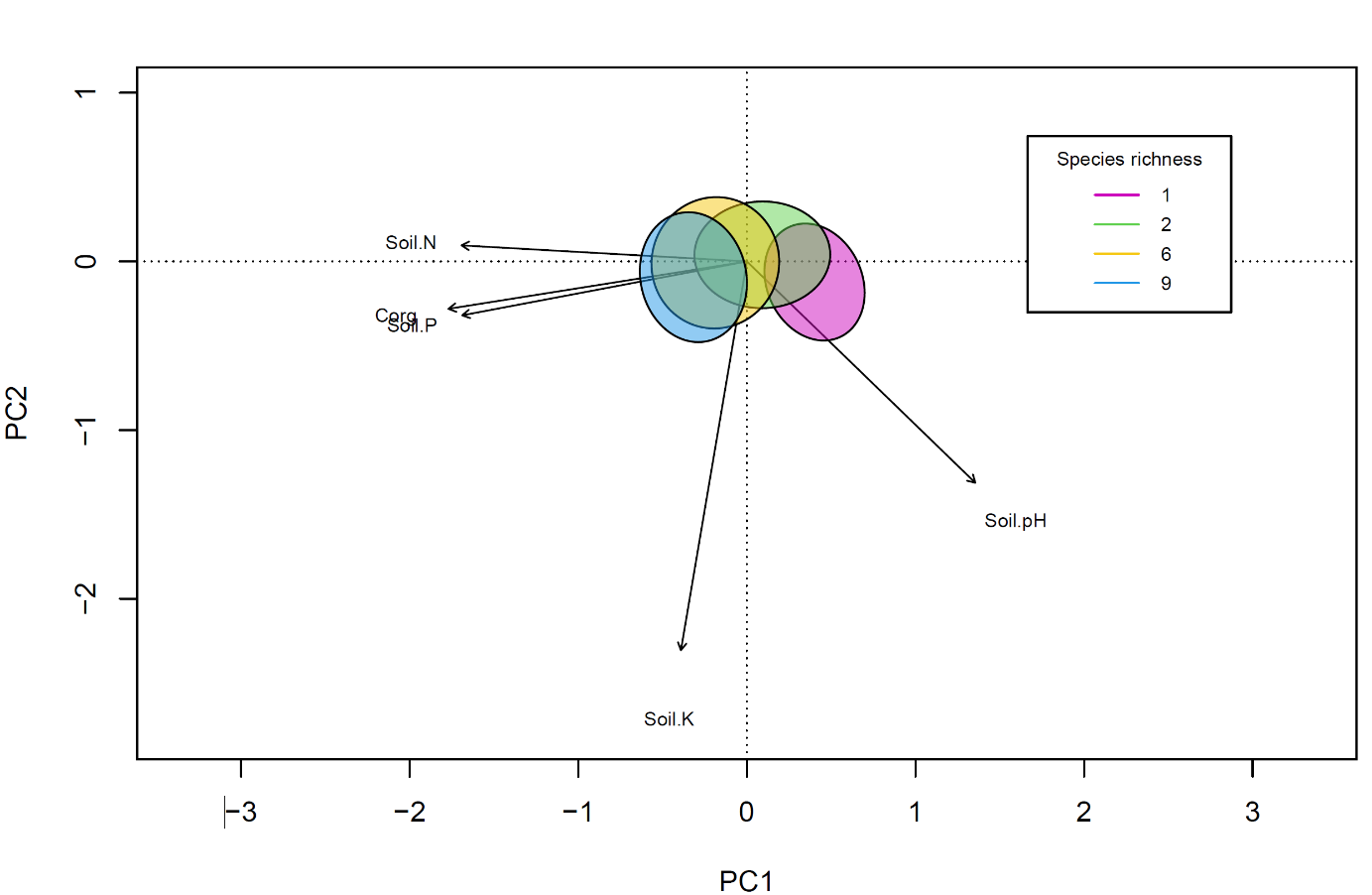
**

**Figure S5** Variance partitioning analysis depicting the proportion of variance in community biomass production (a), net biodiversity effects (b), selection effects (c), and complementarity effects (d) explained by plant traits (plant PC1 and PC2 derived from plant PCA), soil properties (soil PC1 and PC2 derived from soil PCA) and sown plant species richness.

**
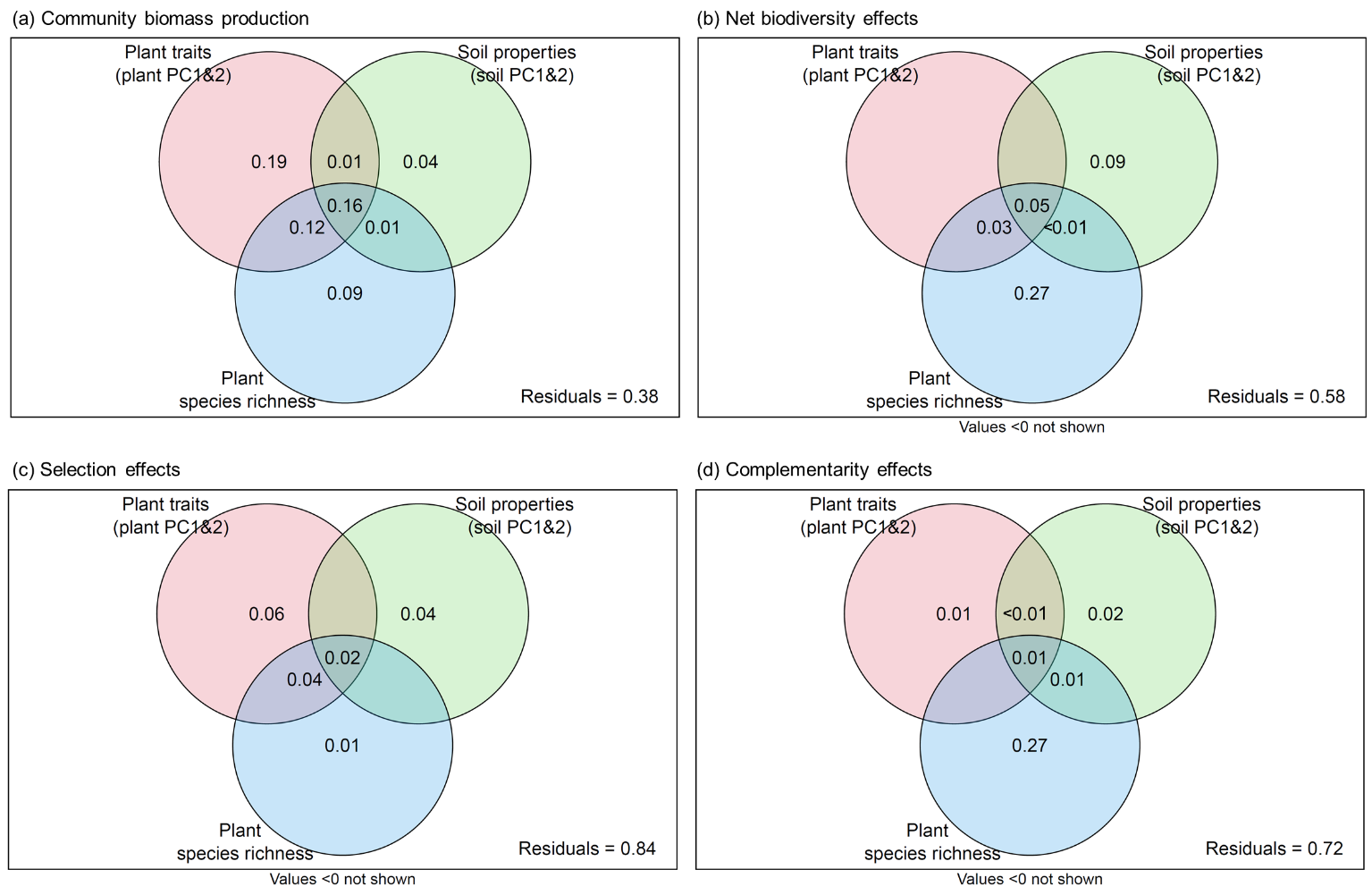
**
